# Metaproteomic analysis of ratoon sugarcane rhizospheric soil

**DOI:** 10.1186/1471-2180-13-135

**Published:** 2013-06-17

**Authors:** Wenxiong Lin, Sheng Lin, Aijia Zhang, Mingming Zhou, Rui Lin, Haibin Wang, Jun Chen, Zhixing Zhang, Ruiyu Lin

**Affiliations:** 1 Key Laboratory of Biopesticide and Chemical Biology, Ministry of Education, Fujian Agriculture and Forestry University, Fuzhou 350002, Fujian, P. R. China; 2 Agroecological Institute, Fujian Agriculture and Forestry University, Fuzhou 350002, Fujian, P. R. China; 3 College of Oceanography and Environmental Science, Xiamen University, Xiamen 361005, Fujian, P. R. China

**Keywords:** CLPP, 2D-electrophoresis, Soil enzyme, Soil metaproteomics, Soil protein extraction, Sugarcane

## Abstract

**Background:**

The current study was undertaken to elucidate the mechanism of yield decline in ratoon sugarcane using soil metaproteomics combined with community level physiological profiles (CLPP) analysis.

**Results:**

The available stalk number, stalk diameter, single stalk weight and theoretical yield of ratoon cane (RS) were found to be significantly lower than those of plant cane (NS). The activities of several carbon, nitrogen and phosphorus processing enzymes, including invertase, peroxidase, urease and phosphomonoesterase were found to be significantly lower in RS soil than in NS soil. BIOLOG analysis indicated a significant decline in average well-color development (AWCD), Shannon’s diversity and evenness indices in RS soil as compared to NS soil. To profile the rhizospheric metaproteome, 109 soil protein spots with high resolution and repeatability were successfully identified. These proteins were found to be involved in carbohydrate/energy, amino acid, protein, nucleotide, auxin and secondary metabolisms, membrane transport, signal transduction and resistance, etc. Comparative metaproteomics analysis revealed that 38 proteins were differentially expressed in the RS soil as compared to the control soil or NS soil. Among these, most of the plant proteins related to carbohydrate and amino acid metabolism and stress response were up-regulated in RS soil. Furthermore, several microbial proteins related to membrane transport and signal transduction were up-regulated in RS soil. These proteins were speculated to function in root colonization by microbes.

**Conclusions:**

Our experiments revealed that sugarcane ratooning practice induced significant changes in the soil enzyme activities, the catabolic diversity of microbial community, and the expression level of soil proteins. They influenced the biochemical processes in the rhizosphere ecosystem and mediated the interactions between plants and soil microbes.

## Background

Sugarcane (*Saccharum* L. spp. hybrids) is of tremendous economic importance not just for the sugar industry but also for its impact on sustainable energy production. The ratoon sugarcane is the regenerated crop plant from the germinating bud of the stubble from the previous crop [1]. Ratooning practice saves cost on preparatory tillage and planting material and benefits from the residual manure and moisture. In addition, the ratoon sugarcane matures earlier than the newly planted sugarcane (plant sugarcane). However, there is a decline in the yield of ratoon sugarcane in the successive years under normal conditions [2]. This has become one of the major problems in the high-yielding cultivation of sugarcane.

The expansion of crop monoculture has led to the simplification of the agroecosystem, and the loss and fragmentation of habitat [3]. Large-scale monocultures result in a decline in the biological diversity, destroy the capability of self-adjustment of the ecosystem, and cause diseases, which further increases the production cost and pollute the environment because more pesticides and better fertilizers are required [3]. The yield decline has been defined as the loss of productive capacity of sugarcane soils under long-term monocultures [4]. Gascho et al. [5] found that productivity of the ratoon sugarcane was 33 percent less than the plant sugarcane due to increased mortality of stalks, reduction in soil nutrition status and abundance of pests and diseases in the soil. Current evidences suggests that several factors (including the long-term sugarcane monoculture, excessive tillage and mechanical harvesting and haul-out with heavy machinery, etc.) are responsible for the degradation of physical, chemical and microbial properties of sugarcane growing soils [6,7]. Recent studies have revealed that crop rotation breaks and organic amendments greatly influence the structure and microbial populations of the sugarcane rhizospheric soil [2,8,9]. Our previous study showed that ratooning cane, intercropped with legumes, enhanced the functional diversity of rhizospheric microbial community and increased cane yield (Data not shown). Plant-soil organism interactions, especially plant-microbial interactions play crucial roles in soil quality, and crop health and yield [10,11]. There has been an increasing interest in the biological properties of rhizosphere *in situ*[12]. However, there is no report hitherto focusing on the relationship among the soil ecosystem, soil organism community and sugarcane ratooning practice from a proteomic perspective.

Various DNA-dependent strategies, such as terminal restriction fragment length polymorphism [13], denaturing gradient gel electrophoresis [14] and reverse transcription-polymerase chain reaction [15] have been used to elucidate the biological information from microbial communities in the soil ecosystem. However, since the mRNA expression and protein expression do not always correlate directly, the function of microbial diversity still remains unknown [16]. Moreover, the biological processes in rhizosphere soil are not only driven by the microbes but also by the plants and the fauna in the ecosystem [17]. Extended soil protein identification is essential for understanding the soil ecological processes and the environmental factors that affect the functioning of the rhizospheric soil ecosystem [18,19]. Two community-based measurements, community level physiological profiles (CLPP) and soil metaproteomics were used in this work. The assessment of microbial functional diversity by using BIOLOG sole carbon (C) substrate utilization tests is a rapid, sensitive approach to detect modifications in diversity due to soil management, disturbance, stress or succession [20]. Soil rhizospheric metaproteomics is a powerful scientific tool to account for functional gene expression in microbial ecosystems and can uncover the interactions between plants and soil microorganisms [17].

It was speculated that the yield decline in ratoon sugarcane is closely related to the dynamics and genetic diversity of the community members (i.e., bacteria, fungi and fauna). Therefore, in this study, we aimed to: (i) determine differences between the soil protein abundance in plant sugarcane and ratoon sugarcane rhizospheric soils, and (ii) analyze interactions between the root system and the rhizospheric soil organisms based on our data from soil enzyme assays, CLPP analysis, and a limited number of identified soil proteins.

## Results

### Sucrose content and theoretical production

The available stalk number per hectare, stalk diameter, single stalk weight and theoretical production of plant cane were found to be significantly (*P* ≤ 0.05) higher than those of ratoon cane. However, there were no significant differences in the sucrose content and stalk height of the 2 types of cane (Table 1).

**Table 1:** **The agronomic characters**, **theoretical sugar content and yield of plant cane and ratoon cane**

	**Sucrose content (%)**	**Available stalk number (hm**^**-2**^**)**	**Stalk height (cm)**	**Stalk diameter (cm)**	**Single stalk weight (kg)**	**Theoretical production (kg/hm**^**2**^**)**
Plant cane	12.86±0.63a	67311.06±555.17a	312.0±1.53a	2.97±0.009a	1.96±0.02a	131785.5±393.7a
Ratoon cane	13.59±0.36a	61541.54±826.24b	325.3±9.17a	2.77±0.066b	1.78±0.10b	109404.8±6641.4b

Note: Data are means ± SE. Different letters in columns show significant differences determined by Tucky’s test (*P* ≤ 0.05).

### Soil enzyme activity

Except for polyphenol oxidase, the other enzymes, i.e. invertase, urease, phosphomonoesterase and peroxidase activities were found to be significantly higher in plant cane soil, than in ratoon cane soil or control soil. There were no significant differences in invertase and peroxidase activities between the control and ratoon cane soils. However, the control soil had significantly lower urease and phosphomonoesterase activities than ratoon cane soil (Table 2).

**Table 2:** Soil enzyme activities in rhizospheric soils from the study sites

	**Invertase **^**a**^	**Urease **^**b**^	**Phosphomonoesterase **^**c**^	**Polyphenol oxidase **^**d**^	**Peroxidase **^**d**^
Control soil	0.21±0.034b	0.020±0.0009c	0.12±0.0091c	0.85±0.074a	1.91±0.101b
Plant cane soil	0.62±0.033a	0.047±0.0023a	0.41±0.0042a	1.18±0.074a	2.50±0.208a
Ratoon cane soil	0.33±0.020b	0.038±0.0013b	0.27±0.0108b	0.88±0.164a	1.88±0.024b

Note: Data are means ± SE. Different letters in columns show significant differences determined by Tucky’s test (*P* ≤ 0.05). ^a^ μmol glucose g^-1 ^soil h^-1^; ^b ^μmol urea g^-1^ soil h^-1^; ^c ^μmol p-nitrophenol g^-1 ^soil h^-1^; ^d ^μmol pyrogallol g^-1 ^soil h^-1^.

### Microbial community dynamics assessed by BIOLOG analysis

**Figure 1:**
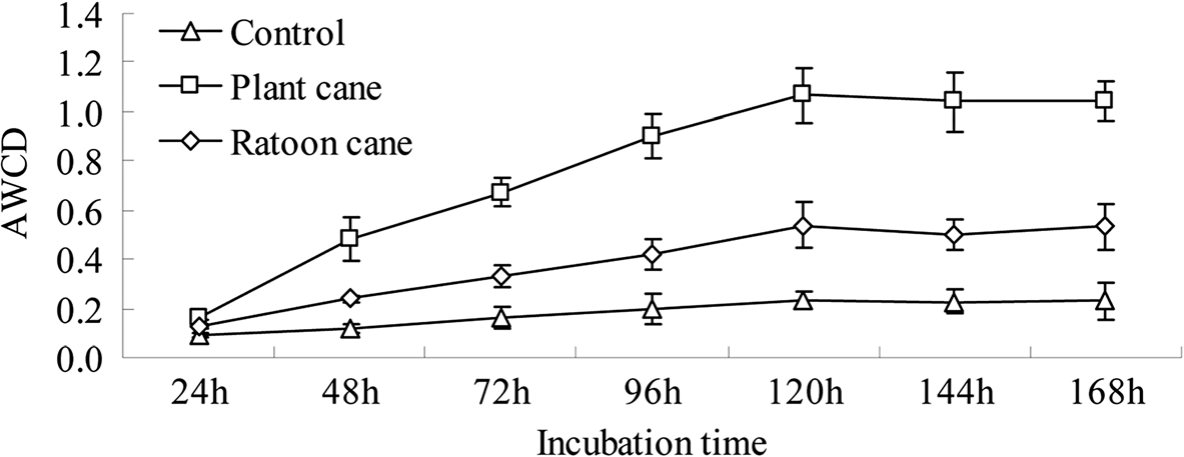
Average well color development (AWCD) of substrate utilization patterns in BIOLOG ECO microplates.

The average well-color development (AWCD) of the carbon substrates for all soil samples using the BIOLOG ECO microplates indicated that the change in AWCD increased with an increase in incubation time during the 168 h incubation period (Figure 1). The AWCD followed the sequence, plant cane (NS) > ratoon cane (RS) > control (CK), at almost every time point monitored. Plant cane soil showed the largest rates of substrate utilization while ratoon cane soil displayed significantly lower rates. Furthermore, CLPP diversity and evenness, evaluated with the data from 96 h incubation, were found to be significantly lower in ratoon cane soil than in plant cane soil. Both, Shannon’s diversity and evenness indices were found to be the lowest in the control soil (Table 3). The consumption of carbohydrates, amines, amino acids and phenolic compounds was significantly reduced in ratoon cane soil compared to that in plant cane soil (Table 3). We found that phenolic compounds were mainly expended in control soil; carbohydrates and amines in plant cane soil; while carboxylic acids and amino acids were expended in ratoon cane soil.

**Table 3:** **Diversity and evenness indices**, **and mean optical density of grouped substrates** (**six groups**) **at 96 h incubation time in different treatments**

	**Control soil**	**Plant cane soil**	**Ratoon cane soil**	***P *values**
Shannon’s diversity index	4.190±0.03c	4.393±0.01a	4.273±0.02b	0.0003
Shannon’s evenness	0.85±0.01b	0.89±0.01a	0.85±0.01b	0.001
Mean OD	0.20±0.06c	0.90±0.09a	0.42±0.06b	0.0001
Polymers	0.12±0.03b	0.37±0.07a	0.30±0.08a	0.008
Carbohydrates	0.18±0.02b	1.31±0.12a	0.28±0.03b	0.0001
Carboxylic acids	0.10±0.04b	0.70±0.15a	0.65±0.08a	0.0007
Amino acids	0.20±0.05c	0.81±0.11a	0.59±0.07b	0.0003
Amines	0.11±0.02b	1.16±0.08a	0.12±0.03b	0.0001
Phenolic compounds	0.84±0.05a	0.53±0.03b	0.39±0.02c	0.0001

Note: Data are means ± SD. Different letters in rows show significant differences determined by Tucky’s test (*P *≤ 0.05).

**Figure 2:**
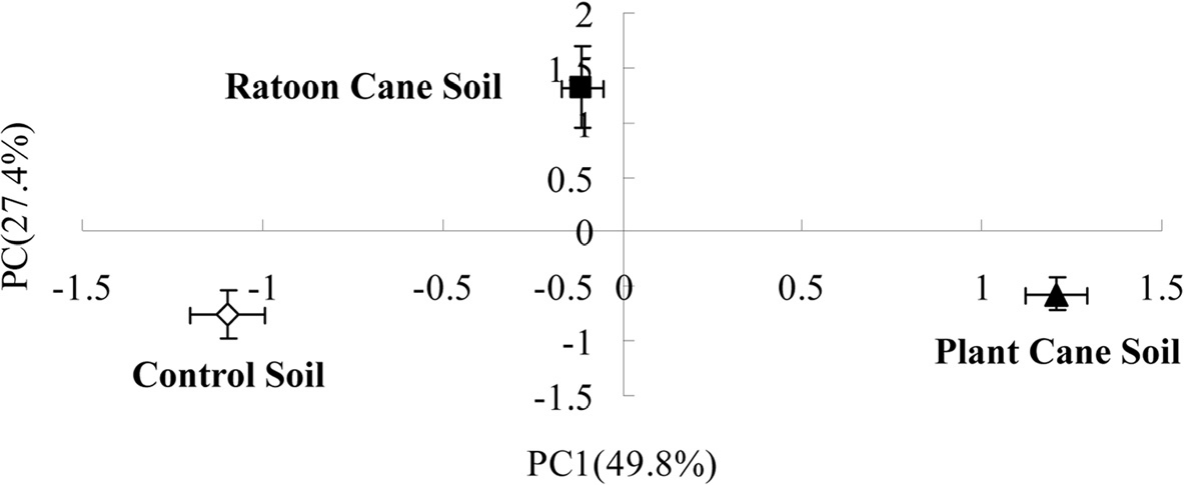
Principal component analysis of substrate utilization patterns from three different rhizospheric soil samples.

Principal component analysis (PCA) indicated that 96 h AWCD data successfully distinguished the response of the 3 soil communities to the carbon substrates (Figure 2). The first principal component (PC1) accounted for 49.8% of the total variation in the ECO microplate data, while PC2 accounted for 27.4% of the total variation in the ECO microplate data. The eight carbon substrates with the most positive and most negative scores (i.e., contributing most strongly to the separation of samples) on PC1 and PC2 are listed in Additional file 1: Table S1. α-Ketobutyric acid and D-glucosaminic acid were discriminated most positively by PC1 scores, while L-asparagine and D-galacturonic acid were discriminated most positively by PC2 scores. However, i-erythritol and glucose-1-phosphate were discriminated most negatively by PC1 scores, while D-galactonic acid γ-lactone and 4-hydroxy benzoic acid were discriminated most negatively by PC2 scores.

### Profile analysis of metaproteome in rhizospheric soils

Approximately 759, 788, and 844 protein spots were detected on silver-stained gel of proteins extracted from the control soil, plant cane soil, and ratoon cane soil respectively (Additional file 2: Figure S1). Highly reproducible 2-DE maps were obtained from the three different soil samples with significant correlations among scatter plots. The correlation index between the control soils and the newly planted sugarcane soils was found to be 0.868, and the correlation index between the control soils and the one year ratoon sugarcane soils were was 0.761.

To obtain a metaproteomic profile for the sugarcane rhizospheric soil, 143 protein spots with high resolution and repeatability, including all 38 differentially expressed proteins and 105 constitutively expressed proteins, were selected for identification and 109 protein spots were successfully analyzed by MALDI TOF-TOF MS (Additional file 3: Figure S2; Additional file 4: Table S2).

**Figure 3:**
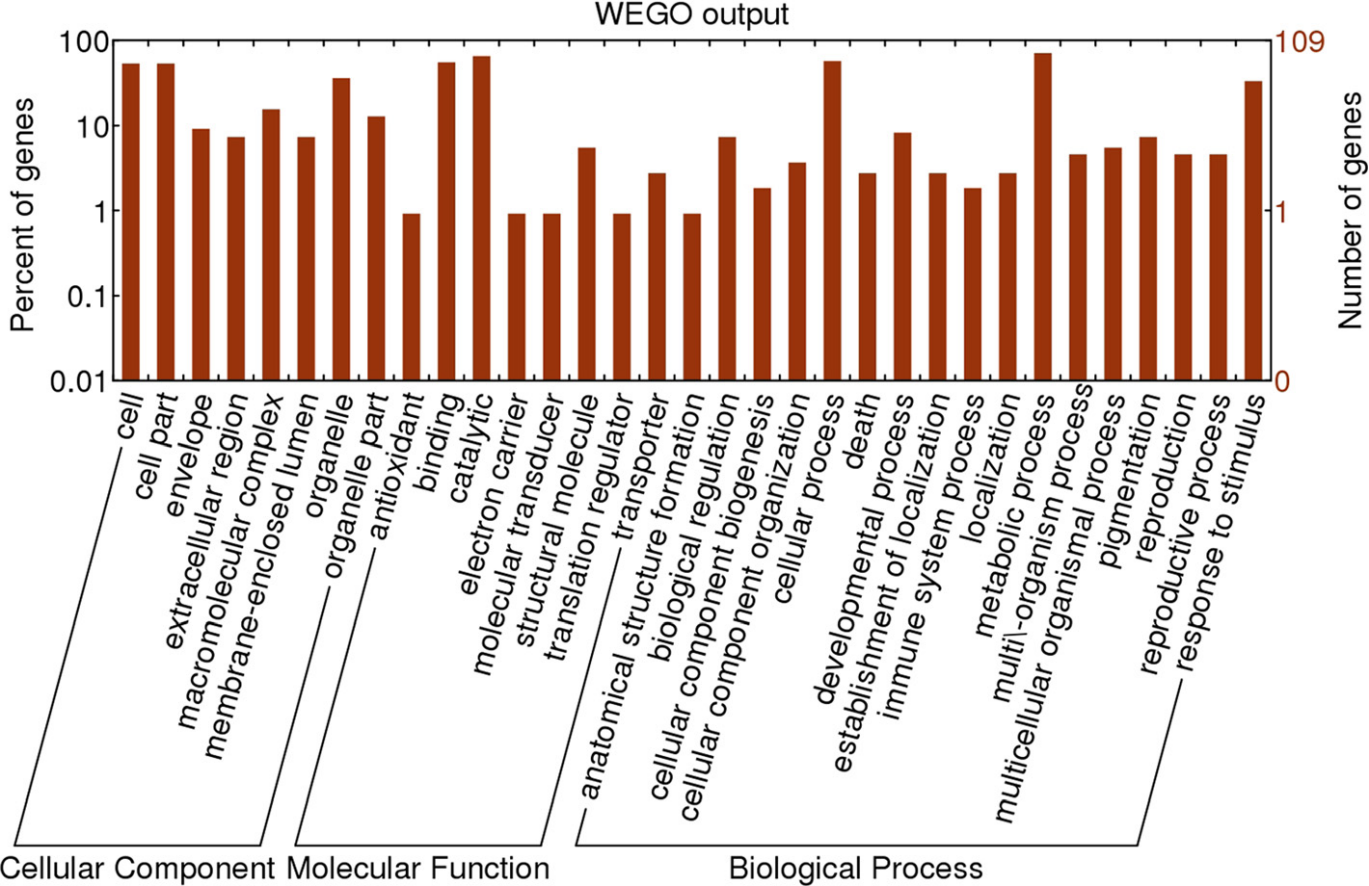
**Gene Ontology (GO) for the identified soil proteins. **The right coordinate axis indicates the number of proteins for each GO annotation, and the left one represents the proportion of proteins for every GO annotation.

According to Gene Ontology (GO) annotations, the identified proteins were classified into 8 Cellular Component (CC), 8 Molecular Function (MF) and 17 Biological Process (BP) categories, as shown in Figure 3. Highly represented categories were associated with ‘cell part’ (53.2% of the GO annotated proteins) and ‘organelle’ (35.8%) in CC, ‘catalytic activity’ (65.1%) and ‘binding’ (55.0%) in MF, ‘metabolic process’ (70.6%), ‘cellular process’ (56.9%) and ‘response to stimulus’ (33.0%) in BP.

**Figure 4:**
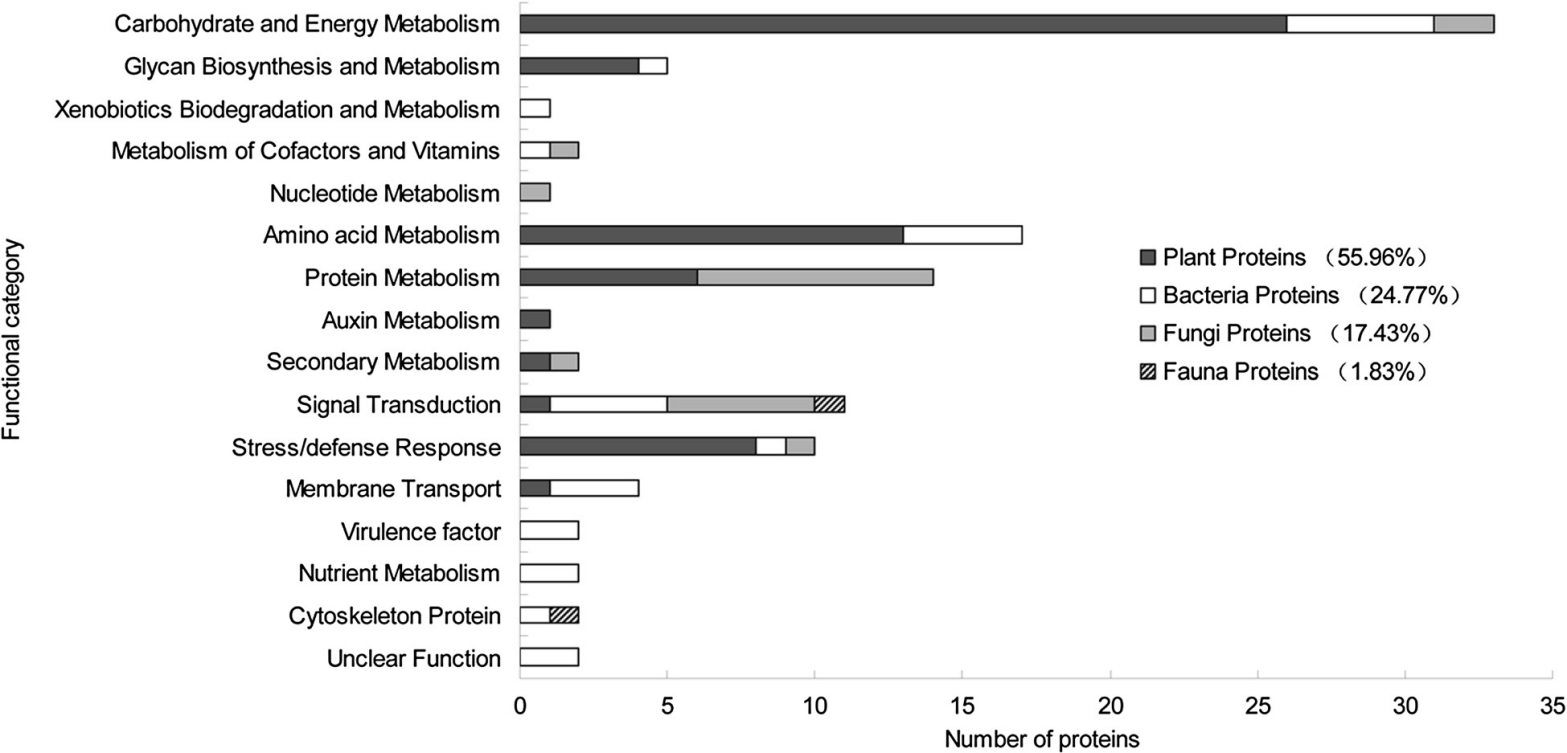
**Functional classification of the identified proteins. **Identified proteins were classified according to their functions using KEGG database (Kyoto Encyclopedia of Genes and Genomes, http://www.genome.jp/kegg/).

According to the putative physiological functions assigned using the KEGG database, these soil proteins were categorized into 16 groups as shown in Figure 4. Among these, 55.96% were derived from plants, 24.77% from bacteria, 17.43% from fungi and 1.83% from fauna (Additional file 4: Table S2). Most of these identified proteins were associated with the carbohydrate/energy metabolism (constituting 30.28%), amino acid metabolism (constituting 15.60%) and protein metabolism (constituting 12.84%). Besides, ten proteins (constituting 9.17%, including the heat shock protein 70 and catalase, etc.) were found to be involved in stress defense and eleven proteins (constituting 10.09%, including the two-component system sensor kinase, G-protein signaling regulator and annexin protein, etc.) relating to the signal transduction were detected (Additional file 4: Table S2). Based on the metaproteomic data, a tentative metabolic model for the rhizospheric soil proteins was proposed as shown in Additional file 5: Figure S3. These soil proteins function in carbohydrate/energy, nucleotide, amino acid, protein, auxin metabolism and secondary metabolism, membrane transport, signal transduction and resistance, etc.. Most of the plant proteins identified, were thought to participate in carbohydrate and amino acid metabolism, which might provide the necessary energy and precursor materials for the organic acid efflux and rhizodeposition process, defense responses and secondary metabolism under biotic and abiotic stresses. However, some microbial proteins related to the membrane transport (including the ABC transporter ATP-binding subunit, and sugar ABC transporter) and signal transduction (including the two-component system sensor kinase) were also identified in the rhizospheric soil, which might play an important role in the root colonization of microbes. These soil proteins probably influence the rhizodeposition process and mediate the interactions between the plants and the soil organisms.

### Differentially expressed proteins and their roles in rhizospheric soils

As shown in Table 4, the quantitative analysis revealed that a total of 38 protein spots (spots 1-38) with high repeatability displayed differential expression by more than 1.5-fold at least on one gel in comparison to the control [21]. These differentially expressed proteins originated from plants (constituting 50%), bacteria (constituting 34.21%), fungi (constituting 13.16%) and fauna (constituting 2.63%) (Table 4).

**Table 4:** **Differentially expressed proteins identified by MALDI TOF**-**TOF MS**

**Spot no. **^***a*)**^	**GI no. **^***b*)**^	**Protein name**	**Score (PMF) **^***c*)**^	**PMF/Coverage**^***d*)**^	**MW/ p*I ***^***e*)**^	**Score (MS-MS) **^***f*)**^	**Pept **^***g*)**^	**Species**	**Function **^***h*)**^	**RS/** **CK **^***i*)**^	**RS/** **NS **^***j*)**^
12	gi|115470493	Succinate dehydrogenase [ubiquinone] flavoprotein subunit, mitochondrial	106	20/34%	69494/6.61	185	3	*Oryza sativa*	TCA	1.9	1.9
13	gi|115467370	Phosphofructokinase	130	18/38%	61907/6.01	251	4	*Oryza sativa*	EMP	1.7	1.7
16	gi|115459078	Glyceraldehyde-3-phosphate dehydrogenase, cytosolic 3	117	14/51%	36921/6.34	122	2	*Oryza sativa*	EMP	1.6	1.5
18	gi|115480019	Proteasome beta type-1	136	11/50%	24608/6.43	92	2	*Oryza sativa*	Protein degradation	0.8	1.5
23	gi|51090388	Putative PrMC3	107	16/59%	34540/5.61	296	3	*Oryza sativa*	Stress/defense response	1.6	1.7
25	gi|115111257	Betaine aldehyde dehydrogenase	86	10/31%	55361/5.29	276	4	*Oryza sativa*	Amino acid metabolism	2.2	2.2
26	gi|115464537	2,3-bisphosphoglycerate-independent phosphoglycerate mutase	127	20/42%	61003/5.25	361	5	*Oryza sativa*	EMP	2.0	1.0
27	gi|115448989	Heat shock 70 kDa protein, mitochondrial precursor	96	19/34%	73081/5.49	456	4	*Oryza sativa*	Stress/defense response	2.3	2.2
28	gi|54606800	NADP dependent malic enzyme	84	24/37%	65824/5.79	193	3	*Oryza sativa*	Pyruvate metabolism	2.1	2.1
29	gi|115477952	Cyclase family protein	80	11/39%	29792/5.32	115	2	*Oryza sativa*	Signal transduction	2.4	1.0
31	gi|115440691	2,3-bisphosphoglycerate-independent phosphoglycerate mutase	189	30/50%	60980/5.42	500	4	*Oryza sativa*	EMP	1.1	1.7
32	gi|108708038	Fumarate hydratase 1, mitochondrial precursor, putative, expressed	124	13/27%	53991/6.93	210	4	*Oryza sativa*	TCA	1.8	1.6
35	gi|968996	Glyceraldehyde-3-phosphate dehydrogenase	139	14/50%	36641/6.61	379	3	*Oryza sativa*	EMP	1.7	1.5
37	gi|3024122	S-adenosylmethionine synthase 2	100	18/60%	43330/5.60	405	4	*Oryza sativa*	Amino acid metabolism	0.4	0.6
1	gi|1203832	Beta-D-glucan exohydrolase, isoenzyme ExoII			67835/7.96	153	2	*Hordeum vulgare*	Glycan metabolism	4.0	1.5
4	gi|3868754	Catalase			57052/6.49	147	2	*Oryza sativa*	Stress/defense response	2.9	1.7
21	gi|115455455	UDP-glucose 6-dehydrogenase			53435/5.79	208	3	*Oryza sativa*	Glycan metabolism	1.5	1.0
33	gi|38605779	NAD-dependent isocitrate dehydrogenase			36882/5.77	221	3	*Oryza sativa*	TCA	1.8	1.0
2	gi|226357624	Putative sugar ABC transporter, periplasmic component	84	10/33%	46676/9.68			*Deinococcus deserti*	Membrane transport	3.0	1.6
3	gi|241957693	Mitochondrial N-glycosylase/DNA lyase	74	11/39%	40573/8.46			*Candida dubliniensis*	Nucleotide metabolism	3.1	1.9
5	gi|254399905	ABC transporter ATP-binding subunit	82	18/31%	66963/5.53			*Streptomyces sviceus*	Membrane transport	2.0	1.5
6	gi|126662203	Oxidoreductase	74	13/20%	76867/8.83			*Flavobacteria bacterium*	Oxidation reduction	2.4	1.7
7	gi|261195979	ORP1	74	10/39%	36747/9.48			*Ajellomyces dermatitidis*	Signal transduction	1.6	1.5
8	gi|238481813	ADP-ribosylglycohydrolase	84	18/28%	49119/6.02			*Aspergillus flavus*	Signal transduction	1.0	0.5
9	gi|261854741	Phosphoribosylformimino-5-aminoimidazole carboxamide ribotide isomerase	85	9/41%	26805/4.63			*Halothiobacillus neapolitanus*	Amino acid metabolism	0.6	0.6
10	gi|115456914	Elongation factor EF-2	101	23/31%	94939/5.85			*Oryza sativa*	Protein metabolism	4.6	2.3
11	gi|219667596	Radical SAM domain protein	82	11/46%	38272/5.24			*Desulfitobacterium hafniense*	Diverse reaction	2.3	2.5
14	gi|111024023	Acyl-CoA dehydrogenase	87	13/37%	41071/5.40			*Rhodococcus jostii*	Amino acid metabolism	2.8	1.9
15	gi|23009750	Succinate dehydrogenase/fumarate reductase, Fe-S protein subunit	87	7/92%	6114/4.52			*Magnetospirillum magnetotacticum*	TCA	1.9	1.0
17	gi|253988359	Phosphoglycerate kinase	83	9/33%	41652/5.19			*Photorhabdus asymbiotica*	EMP	0.6	1.0
19	gi|94497581	Electron-transferring-flavoprotein dehydrogenase	84	9/25%	61194/5.66			*Sphingomonas* sp.	Energy metabolism	0.5	0.6
20	gi|85110870	Related to kinesin-like protein	74	26/21%	195364/5.31			*Neurospora crassa*	Cytoskeleton protein	2.0	2.0
22	gi|194366013	Nitrate reductase, alpha subunit	71	19/16%	140507/5.98			*Stenotrophomonas maltophilia*	Nitrogen metabolism	1.9	1.1
24	gi|21492793	Conjugal transfer protein A	91	24/19%	171793/6.93			*Rhizobium etli*	Bacterial conjugation	2.1	1.0
30	gi|219664364	Two-component system sensor kinase	87	19/15%	176010/6.50			*Rhodococcus* sp.	Signal transduction	3.0	1.6
34	gi|126135008	Isocitrate dehydrogenase [NADP], mitochondrial precursor	76	14/32%	48355/8.21			*Pichia stipitis*	TCA	1.7	1.7
36	gi|52426030	MrcA protein	90	18/25%	96552/6.40			*Mannheimia succiniciproducens*	Glycan metabolism	1.6	1.0
38	gi|148685933	Tubulin, gamma complex associated protein 2	90	18/29%	89598/6.52			*Mus musculus*	Cytoskeleton protein	0.6	0.9

Note: Protein spots 12, 13, 16, 18, 23, 25-29, 31, 32, 35 and 37 shared equal searching by MS/MS and MS. Protein spots 1, 4, 21 and 33 matched at least two MS/MS peptides. The remainders matched at least three PMFs. ^a) ^The numbering corresponds to the 2-DE gel in Additional file 3: Figure S3. ^b) ^GI number in NCBI. ^c) ^MASCOT score of PMF. ^d) ^The number of peptides identified by MS/sequence percentage coverage. ^e)^ Theoretical molecular weight and p*I*. ^f) ^MASCOT score of MS/MS. ^g) ^Number of peptides identified by MS/MS. ^h) ^Functional classification using KEGG database. ^*i*) ^the ratio of ratoon cane soil (RS) to control soil (CK). ^j) ^the ratio of ratoon cane soil (RS) to plant cane soil (NS).

**Figure 5:**
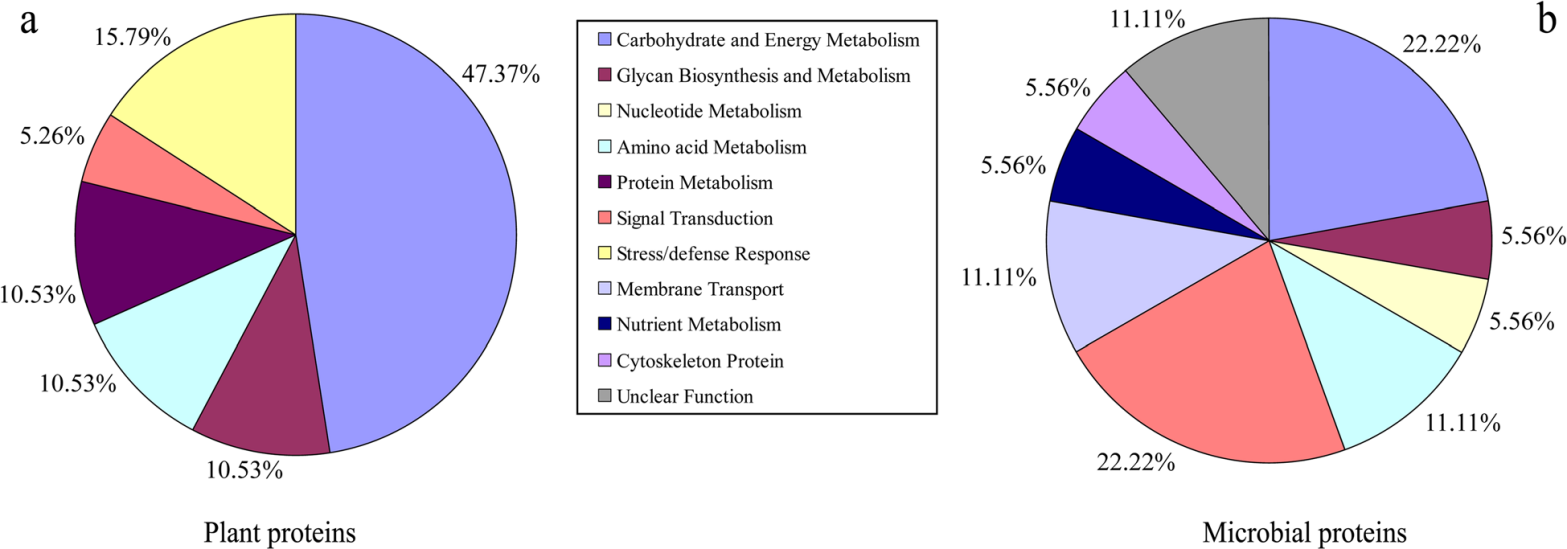
The functional category distribution of differentially expressed proteins originated from the plants (a) and the microbes (b).

Among the plant-originating differentially expressed proteins, the largest functional group found was of the proteins involved in carbohydrate and energy metabolism (constituting 47.37%), followed by those associated with stress/defense response (constituting 15.79%) (Figure 5). Furthermore, most of plant proteins related to carbohydrate/energy metabolism (including spot 12, succinate dehydrogenase; spot 13, phosphofructokinase; spots 16 and 35, glyceraldehyde-3-phosphate dehydrogenase; spot 28, NADP dependent malic enzyme and spot 32, fumarate hydratase 1) and amino acid metabolism (i.e. spot 25, betaine aldehyde hydrogenase) were found up-regulated in the ratoon cane soil, compared to the plant cane and control soils (Table 4). These up-regulated plant proteins involved in carbohydrate and amino acid metabolism probably provide the energy necessary and precursor materials for plant root secretion and rhizodeposition process, which serve as a nutrient source for root-associated microbes. Several proteins (including spot 4, catalase; spot 23, PrMC3 and spot 27, heat shock 70 kDa protein) related to plant stress defense were up-regulated in the ratoon cane soil (Table 4).

Among the microbe-originating differentially expressed proteins, most of them were associated with the carbohydrate/energy metabolism (22.22%) and signal transduction (22.22%) (Figure 5). Several microbial proteins were found related to the root-colonizing ability of microorganisms (including spot 30, two-component system sensor kinase) and the utilization of root exudates (including spot 2, sugar ABC transporter and spot 5, ABC transporter ATP-binding subunit) were up-regulated in the ratoon cane soil, as compared to the plant cane and control soil (Table 4), which might be a response of microbes to the rhizodeposition of ratoon cane. Furthermore, most of proteins originated from fungi (including spot 3, mitochondrial N-glycosylase/DNA lyase; spot 7, ORP1; spot 20, kinesin-like protein and spot 34, isocitrate dehydrogenase) were up-regulated in the ratoon cane soil (Table 4). Besides, one cytoskeleton protein (spot 38, i.e. tubulin gamma) originated from the fauna was identified as well. Therefore, sugarcane ratooning induced the alteration of the expression of soil proteins from the plants, microbes and fauna.

## Discussion

The consecutive monocultures for many medicinal plants and crop plants, such as *Rehmannia glutinosa*[22] and soybea [23], etc., result in a significant reduction in the yield and quality of the harvest. This phenomenon is known as soil sickness (replanting disease) [24] or consecutive monoculture problems [25]. In the present study, the available stalk number per hectare, stalk diameter, single stalk weight) and theoretical production of ratoon cane were found to be significantly (*P* ≤ 0.05) lower than those of plant cane (Table 1). Hunsigi [26] indicated that ratooning practice decreased soil fertility under consecutive sugarcane cropping. Several researchers developed a ‘farming systems’ approach to address the problem of sugarcane cultivation with a major focus on the introduction of rotation breaks and organic amendments and found that these practices induced remarkable changes in the commnunity composition and structure of the soil biota (bacteria, fungi and nematodes, etc.) [8,27,28]. Enzyme activity in soil is a measure of the soil microbial activity and plays an important role in nutrient cycles and transformations. Therefore, it is used as an indicator of changes into determine changes in quality and productivity of soil [29,30]. In the present study, five soil enzymes activities involved in nutrition cycling and stress response were assayed. Our data showed that the activities of soil enzymes such as invertase, urease, phosphomonoesterase and peroxidase were significantly lower (*P* < 0.05) in ratoon cane soil than in plant cane soil (Table 2).

The assessment of microbial functional diversity by carbon substrate utilization patterns has been reported to be a sensitive approach to detect variability in metabolic potential due to soil management [31]. In the current work, the BIOLOG results showed that ratooning practice led to significant decreases (*P* < 0.05) in AWCD, Shannon’s diversity, and evenness indices in soil as compared to the plant cane soil (Table 3). Particularly, there were significantly lower levels (*P* < 0.05) of carboxyhydrates, amines and amino acids used in ratoon cane soil than in plant cane soil (Table 3). Principal component analysis allowed the differentiation of ratoon cane soil from the control and the plant cane soil. However, the use of BIOLOG ECO microplates to analyze the metabolic diversity of the microbial community represents only the *in situ* phenomena where only the fast growing microbes are involved, and ignores the catabolic profiles of functionally inactive microorganisms [32]. Preston-Mafham et al. [33] claimed that BIOLOG measurements should be applied in community comparisons rather than in community characterization. The trophic structure and the relationship between its components in soil are still poorly understood as the soil food web and biochemical processes are extraordinarily complex. Comparative metaproteomics was used to study the differences in functional gene expression that are mediated by sugarcane ratooning practice in the rhizosphere ecosystem. These differentially expressed proteins were related to various metabolic pathways such as carbohydrate/energy metabolism, amino acid metabolism, signal transduction, membrane transport, and stress/defense response etc.. These results might help to unravel the intricate interactions among plant root systems, root exudates, and rhizospheric microflora.

### Differentially expressed plant proteins under ratooning practice

Our metaproteomic analysis showed that the 6 proteins (spot 12, succinate dehydrogenase; spot 13, phosphofructokinase; spots 16 and 35, glyceraldehyde-3-phosphate dehydrogenase and spot 32, fumarate hydratase 1) linked to the glycolysis (EMP) / tricarboxylic acid (TCA) cycle and one protein (spot 25, betaine aldehyde hydrogenase) involved in glycine, serine and threonine metabolism were highly expressed in the ratoon cane soil, as compared to the plant cane and control soils (Table 4). These proteins are probably associated with the release of root exudates from plants. Many root exudates (such as malate, fumarate, oxalate, malonate, citrate, aconitate, arginine, histidine and lysine) are mostly the intermediates of the TCA cycle or amino acid metabolism. Singh and Mukerji [34] suggested that these root exudates were the determinants of rhizospheric microbial biodiversity. Root exudates act as chemo-attractants that function to attract bacteria towards roots [35]. The qualitative and quantitative composition of root exudates is affected by various environmental factors (such as pH, soil type, oxygen status, nutrient availability, etc.) and the presence of microorganisms. The up-regulation of these proteins involved in the carbohydrate and amino acid metabolism might be explained by a change in the composition of root exudates possibly resulting from soil disturbances which might be caused by ratooning.

In this study, three proteins linked to plant stress/defense response (including spot 4, catalase; spot 23, PrMC3 and spot 27, heat shock 70 kDa protein) showed higher expression levels in the ratoon cane soil than in the plant cane and control soils (Table 4). Catalase and heat shock protein 70 (Hsp 70) have been proven to be critical for various abiotic and biotic stress responses [36-38]. The above mentioned proteins are rapidly up-regulated in pathogen infection and play a central role in defense against pathogens [39,40]. PrMC3 is a member of a family of proteins that all contain a Ser-hydrolase motif (GxSxG) and is similar to the tobacco protein hsr203J [41]. Hsr203J is rapidly and specifically expressed in the hypersensitive response to various pathogens in tobacco [42]. Furthermore, Zhou et al. [43] found that the gene expression of PrMC3 was up-regulated in the plant leaves infected by the bacterial pathogen *Xanthomonas oryzae* pv. *Oryzicola*. Therefore, the up-regulation of catalase, PrMC3 and Hsp70 might imply that ratoon cane was confronted with environmental stress in the soil, which possibly results from the presence of certain pathogens (pathogenic microbes or root-infecting nematodes) [44,45] or other abiotic stresses in the ratooning system.

### Differentially expressed microbial proteins under ratooning practice

The results from our experiments showed that the two proteins (spots 2, sugar ABC transporter and spot 5, ABC transporter ATP-binding subunit) linked to the membrane transport and one protein (spot 30, two-component system sensor kinase) related to signal transduction had higher expression levels in the ratoon cane soil, as compared to the plant cane and control soils (Table 4). ABC transporters are multicomponent systems, which include one or two integral membrane proteins that constitute the channel across the membrane, an ATP-binding protein that hydrolyzes ATP and drives the transport, and in most cases, an extracellular solute-binding protein [46]. ABC transport systems play an important role in many different aspects of bacterial physiology, facilitating the import of nutrients, and in the extrusion of toxins and antimicrobial agents [47]. Sugar ABC transporters facilitate the transport of a variety of sugars. Some microorganisms utilize highly efficient sugar ABC transporters to survive when substrate concentrations are extremely low [48]. The two-component system sensor kinase (spot 30) was also found to be up-regulated in our study. The two-component system is one of the signal transduction systems in microorganisms that consists of a sensor histidine kinase (SK) and a response regulator (RR). This system responds to a large number of environmental signals [49] and is postulated to play an important role in root colonization [50]. The up-regulation of the proteins involved in membrane transport and signal transduction might be related to the utilization of rhizodeposition by root-associated bacteria. This probably facilitates root colonization by these bacteria. Besides, most of proteins originated from fungi (including spot 3, mitochondrial N-glycosylase/DNA lyase; spot 7, ORP1; spot 20, kinesin-like protein and spot 34, isocitrate dehydrogenase) showed higher expression levels in ratoon cane soil than in the plant cane and control soils (Table 4). The functional gene expression differences in soil microbial communities are probably mediated by a change in the amount and composition of root exudates [51,52].

Despite the limited number of soil proteins identified, our metaproteomic analysis results, combined with soil enzyme assays and CLPP analysis, provide a solid foundation to understand the interactions between the soil organisms and plants in the soil ecosystem. Environmental metaproteomics has been demonstrated to be a useful tool for structural and functional characterization of microbial communities in their natural habitat [53,54], with an increasing improvement in MS performance [55] and soil protein extraction [56]. Metaproteomics is most powerful when combined with metagenomics or when using unmatched metagenomic datasets [57].

## Conclusion

Our experiments revealed that ratooning practice significantly decreased the activity of soil enzymes, catabolic activity, and Shannon’s diversity and evenness indices. The comparative soil metaproteomics revealed that sugarcane ratooning induced changes in the expression levels of soil proteins originated from the plants, microbes and fauna. A majority of up-regulated plant proteins were found to be related to carbohydrate and amino acid metabolism and stress response, while most of up-regulated microbial proteins were involved in membrane transport and signal transduction. In conclusion, sugarcane ratooning practice induced great changes in the soil enzyme activities, the catabolic diversity of microbial community and the expression level of soil proteins. These changes were found to influence the biochemical processes in the rhizosphere ecosystem and mediated the interactions between plants and soil microbes. The soil proteins identified in our study are almost certainly a small part of the diversity of proteins that were expressed by the plants and soil microbial communities. Yet, environmental metaproteomics is a powerful tool to study plant-microbe interactions in soil.

## Methods

### Site description and soil sampling

The sugarcane cultivar ‘Gan-nang’ was used in this study. The study plots were completely randomized and located at the experimental farm (26°09′N, 119°23′E) of Ministry of Agriculture Key Laboratory for Sugarcane Genetic Improvement, Fujian Agriculture and Forestry University, Fuzhou, P. R. China. The first site (defined as ‘RS’) was a field used for ratoon sugarcane cultivation, in which sugarcane was newly planted on February 15, 2009 and then ratooned in 2010. The second site (defined as ‘NS’) was a field kept fallow in 2009 and then used for newly planted sugarcane cultivation on February 15, 2010. The field with no sugarcane crop (bare fallow) during the entire experimental period of 2 years was used as a control (defined as ‘CK’). These three different treatments (‘CK’, ‘NS’ and ‘RS’) were organized within a single field site and under the same field management conditions during the entire experimental period. Three replicates were taken for each treatment. Approximately, 150 grams of soil samples from 3 cultivation patterns were obtained from 5 random locations on each plot in September 15, 2010. Soil sampling of all three treatments was carried out at the same time in order to minimize the effect of year-to-year environmental variability. The plot samples were mixed to make composite samples. The intact roots were carefully uprooted with a forked spade and shaken to remove loosely attached soil. The rhizospheric soil tightly attached to the roots was collected and then sieved through 2 mm mesh to remove plant roots, leaf remains, insects, etc. The fresh soil samples were immediately used for soil enzyme and BIOLOG analysis. For protein extraction, the soil samples were dried at 70°C for 2 h, pulverized in a mortar, and sieved through a 0.45 mm mesh to extract soil proteins.

### Sucrose content and theoretical production determination

The sucrose content was determined by Extech Portable Sucrose Brix Refractometer (Mid-State Instruments, CA, USA) on December 15, 2010 and calculated by using the formula [58]: sucrose (%) = brix (%)×1.0825-7.703. Meanwhile, the theoretical production of sugarcane was calculated according to the following equations [58]: (1) Single stalk weight (kg) = [stalk diameter (cm)]^2^×[stalk height (cm)-30]×1 (g/cm^3^)×0.7854/1000; (2) Theoretical production (kg/hm^2^) = single stalk weight (kg)×productive stem numbers (hm^-2^).

### Soil enzyme assays

The activities of five soil enzymes involved in the cycling of carbon, nitrogen, and phosphorus and stress responses, i.e., invertase (E.C. 3.2.1.26), urease (E.C. 3.5.1.5), acid phosphomonoesterase (E.C. 3.1.3.2), polyphenol oxidase (E.C. 1.10.3.1) and peroxidase (E.C. 1.11.1.7) were determined immediately from freshly sampled soil. Invertase and urease activities were measured following the method of Wang et al. [59] with 8% sucrose and 10% urea (w/v) as substrates, respectively. Acid phosphomonoesterase was assayed with 50 mM p-nitrophenyl phosphate (PNP) as substrate according to the method of Carine et al. [60]. Polyphenol oxidase and peroxidase activities were determined as described by Yu et al. [61] using 1% pyrogallic acid as substrate. Three replicates for each soil sample were taken to perform enzyme assays.

### BIOLOG analysis

Community level physiological profiles (CLPP) were assessed by the BIOLOG Eco MicroPlate™ system (Biolog Inc., CA, USA) according to the method of Lin et al. [62]. Three technical replicates were performed for each treatment. The plates were incubated at 25°C for 168 h, and the color development in each well was recorded as optical density (OD) at 590 nm with a plate reader (Thermo Scientific Multiskan MK3, Shanghai, China) at regular 24 h-intervals.

Microbial activity in each microplate, expressed as average well-color development (AWCD) was determined as follows: AWCD = ∑(*C*-*R*)/31, where *C* is the optical density within each well, *R* is the absorbance value of the plate control well. The 31 carbon substrates in ECO microplates were subdivided into six categories (polymers, carbohydrates, carboxylic acids, amino acids, amines and phenolic compounds) following Choi et al.’s method [63]. The optical density at 96 h incubation time was used to calculate diversity and evenness indices as well as principal component analysis [62], since it was the shortest incubation time that provided the best resolution for all treatments [20].

### Protein extraction and purification

The soil proteins from cultivated samples were extracted and purified by the following protocol developed in our lab [17]. Briefly, 1 g of dry cultivated soil powder were extracted using 5 mL of 0.05 M citrate buffer (pH 8.0) and 5 mL of 1.25% SDS buffer (1.25% w/v SDS, 0.1 M Tris-HCl, pH 6.8, 20 mM DTT), respectively. Citrate extract and SDS extract were shaken for 30 min with 2 mL of buffered phenol (pH 8.0). The two phases were separated by centrifugation for 30 min at 12 000 rpm at 4°C. The proteins in the lower phenol phase were precipitated with 6-fold volume of 0.1 M ammonium acetate dissolved in methanol at -20°C for 6 h. Proteins were recovered by centrifugation for 25 min at 12 000 rpm at 4°C. The pellet was washed once with cold methanol and twice with cold acetone. The washed pellets obtained from citrate extraction and SDS extraction were mixed, air-dried and stored at -80°C until further use.

### 2D-polyacrylamide gel electrophoresis (2D-PAGE) of extracted proteins

The protein pellets were dissolved in appropriate lysis solution (7 M urea, 2 M thiourea, 65 mM DTT, 4% CHAPS, 0.05% v/v ampholytes pH 3.5-10). Protein concentration was determined by Bradford assay using dilutions of bovine serum albumin as standards. 2-D gel electrophoresis (2-DE) was performed as described by Wang et al. [17]. The prepared protein samples were separated by isoelectric focusing (IEF, pH 5–8) in the first dimension, and SDS-PAGE (5% acrylamide stacking gel and a 10% acrylamide separating gel) in the second dimension. After electrophoresis, 2-DE gels were stained with silver nitrate [64]. The gels were scanned using the Image Master (version 5.0, GE Healthcare, Uppsala, Sweden) and analyzed with ImageMaster™ 2D Platinum software (version 5.0, GE Healthcare, Uppsala, Sweden). Repeatability analysis of 2-DE maps of soil proteins was carried out through scatter plots with ImageMaster™ 2D Platinum according to the manufacturer’s instructions. To compensate for subtle differences in sample loading, gel staining, and destaining, the volume of each spot (i.e., spot abundance) was normalized as a relative volume, that is, the spot volume was divided by the total volume over the whole set of gel spots. Standard deviation (SD) was calculated from spots of the gels from three independent experiments and used as error bars. Only those with significant and reproducible changes were considered to be differentially expressed proteins (differing by > 1.5-fold).

### MALDI-MS and protein identification

The interesting protein spots were excised manually from gels for mass spectrometric analysis and the in-gel digestion of proteins were performed as described by Wang et al. [17]. Thereafter, 1 μl of the abovementioned solution was spotted onto stainless steel sample target plates. Peptide mass spectra were obtained on a Bruker UltraFlex III MALDI TOF/TOF mass spectrometer (Bruker Daltonics, Karlsruhe, Germany). Data were acquired in the positive MS reflector mode using 6 external standards for the instrument calibration (Peptide Calibration Standard II, Bruker Daltonics). Mass spectra were obtained for each sampled spot by accumulating of 600-800 laser shots in an 800-5,000 Da mass range. For the MS/MS spectra, 5 most abundant precursor ions per sample were selected for subsequent fragmentation, and 1,000-1,200 Da laser shots were accumulated per precursor ion. The criterion for precursor selection was a minimum S/N of 50.

BioTools 3.1 and the MASCOT 2.2.03 search engine were used to automate database searching. Both MS/MS and MS data were used for the identification of proteins with the following selection criteria: NCBInr database (release 20110409, 13655082 sequences; 4686307983 residues), taxonomy of all entries followed by ‘Bacteria’ or ‘Fungi’ database, trypsin of the digestion enzyme, one missed cleavage site, parent ion mass tolerance at 100 ppm, MS/MS mass tolerance of 0.5 Da, carbamidomethylation of cysteine (global modification), and methionine oxidation (variable modification). The probability score (95% confidence interval) calculated by the software was used as criteria for correct identification [57].

Due to the vast varieties of soil sample sources, the mass spectra were searched against databases step by step. Firstly, ‘all entries’ in NCBInr, which include all organisms, was selected for the search. Then, the ‘Bacteria’ and ‘Fungi’ databases were separately selected to avoid the failed matching when ‘all entries’ was used. The above strategy alleviated the problem of missing some of the mass spectra for matches in searching against ‘all entries’, and allowed significant matching results by searching against ‘Bacteria’ and ‘Fungi’ databases. Both MS/MS and MS data were used for the identification of proteins. The proteins sharing equal searches by MS/MS and MS were preferentially selected. Furthermore, the proteins that matched at least two MS/MS peptides or six peptide mass fingerprintings (PMFs) were subjected to further identification. Only the proteins with the highest score and similar predicted molecular mass were selected.

### Gene ontology and KEGG pathway analysis

Gene Ontology (GO) annotations for the identified soil proteins were assigned according to those reported in the uniprot database [65]. WEGO (Web Gene Ontology Annotation Plotting) tool [66] was used for plotting GO annotation results at GO level 2 as described by Ye et al. [67]. Furthermore, these proteins were used to search KEGG database [68] to obtain reference pathways.

## Abbreviations

AWCD: Average well-color development; BP: Biological process; CC: Cellular component; CK: Control (with no sugarcane crop); CLPP: Community level physiological profiles; 2-DE: 2D-polyacrylamide gel electrophoresis; GO: Gene Ontology; KEGG: Kyoto Encyclopedia of Genes and Genomes; MF: Molecular function; NS: Plant cane; PC: Principal component; PCA: Principal component analysis; RS: Ratoon cane; SDS-PAGE: Sodium dodecyl sulfate polyacrylamide gel electrophoresis; WEGO: Web Gene Ontology Annotation Plotting.

## Competing interests

The authors declare that they have no competing interests.

## Authors’ contributions

WL participated in the design of the study and corrected the manuscript. LW participated in its design and coordination and drafted the manuscript. LW, SL, AZ and HW participated in the extraction of soil proteins and 2D-PAGE. MZ and RYL participated in the BIOLOG analysis. MZ, JC and RYL participated in the determination of agronomic characters. LW, ZZ and JC participated in the protein identification by MALDI TOF-TOF MS. RL performed the bioinformatics analysis. All authors read and approved the final manuscript.

## Supplementary Material

Additional file 1: Table S1 Most discriminant eight carbon substrates as determined by PCA on the data of community level carbon source utilization using BIOLOG Eco microplates by different soil communities. Click here for file

Additional file 2: Figure S1 Silver stained 2-D gel of proteins extracted from the control soil (a), plant cane soil (b) and ratoon cane soil (c). Arrows in (a) point at proteins with differential expression. Upward arrows in (b) and (c) indicate the positions of up-regulated proteins and downward arrows show the positions of down-regulated proteins, while white circles in (b) and (c) represent the same expression level compared to the control. Click here for file

Additional file 3: Figure S2 Representative 2-DE gel of proteins extracted from the plant cane soil. Spot numbers correspond to numbers used in Additional file 4: Table S2. Click here for file

Additional file 4: Table S2 Soil proteins identified by MALDI TOF-TOF MS. Click here for file

Additional file 5: Figure S3 Proposed metabolic model for rhizosphere soil proteins as inferred by metaproteomic data. Identification numbers (E.C.-.-.-.-.) refer to the identified proteins. Blue Upward arrows indicate the up-regulated proteins and downward arrows show the down-regulated proteins. EMP: Embden-Meyerhof pathway; TCA: tricarboxylic acid cycle; GAC: glyoxylic acid cycle; PPP: pentose phosphate pathway. Click here for file
