# Supplementary material for: Metaproteomic analysis of ratoon sugarcane rhizospheric soil
Source: BMC Microbiol. 2013 Jun 17;13:135. doi: 10.1186/1471-2180-13-135 (PMC3687580; doi:10.1186/1471-2180-13-135)
Supplement: Additional file 1: Table S1 — Most discriminant eight carbon substrates as determined by PCA on the data of community level carbon source utilization using BIOLOG Eco microplates by different soil communities. [file 1471-2180-13-135-S1.doc]

**Table S1. Most discriminant eight carbon substrates as determined by PCA on the data of community level carbon source utilization using Biolog Eco microplates by different soil communities**

| Substrate no. | PC1 | Score | PC2 | Score |
| --- | --- | --- | --- | --- |
| 1 | α-Ketobutyric Acid | 1.111 | L-Asparagine | 2.2983 |
| 2 | D-Glucosaminic Acid | 1.110 | D-Galacturonic Acid | 2.2756 |
| 3 | Phenylethylamine | 1.108 | L-Serine | 2.2635 |
| 4 | D-Cellobiose | 1.108 | γ-Hydroxybutyric Acid | 2.207 |
| 5 | Tween 40 | 1.107 | Tween 80 | 2.1448 |
| 6 | Methyl-D-Glucoside | 1.107 | Itaconic Acid | 2.1242 |
| 7 | N-Acetyl-D-Glucosamine | 1.107 | Glucose-1-Phosphate | 2.0761 |
| 8 | Putrescine | 1.107 | L-Phenylalanine | 1.9998 |
| 1 | i-Erythritol | -0.5567 | D-Galactonic Acid γ-Lactone | -2.0841 |
| 2 | Glucose-1-Phosphate | -0.4871 | 4-Hydroxy Benzoic Acid | -2.0132 |
| 3 | D-Galactonic Acid γ-Lactone | -0.4864 | i-Erythritol | -2.0043 |
| 4 | 2-Hydroxy Benzoic Acid | -0.4489 | L-Arginine | -1.3506 |
| 5 | Itaconic Acid | -0.4468 | Glycyl-L-Glutamic Acid | -0.3714 |
| 6 | γ-Hydroxybutyric Acid | -0.3424 | D-Xylose | -0.2294 |
| 7 | 4-Hydroxy Benzoic Acid | -0.2445 | α-D-Lactose | -0.2187 |
| 8 | L-Serine | -0.2177 | N-Acetyl-D-Glucosamine | -0.2183 |
