# Supplementary figures and images for: Metaproteomic analysis of ratoon sugarcane rhizospheric soil
Source: BMC Microbiol. 2013 Jun 17;13:135. doi: 10.1186/1471-2180-13-135 (PMC3687580; doi:10.1186/1471-2180-13-135)

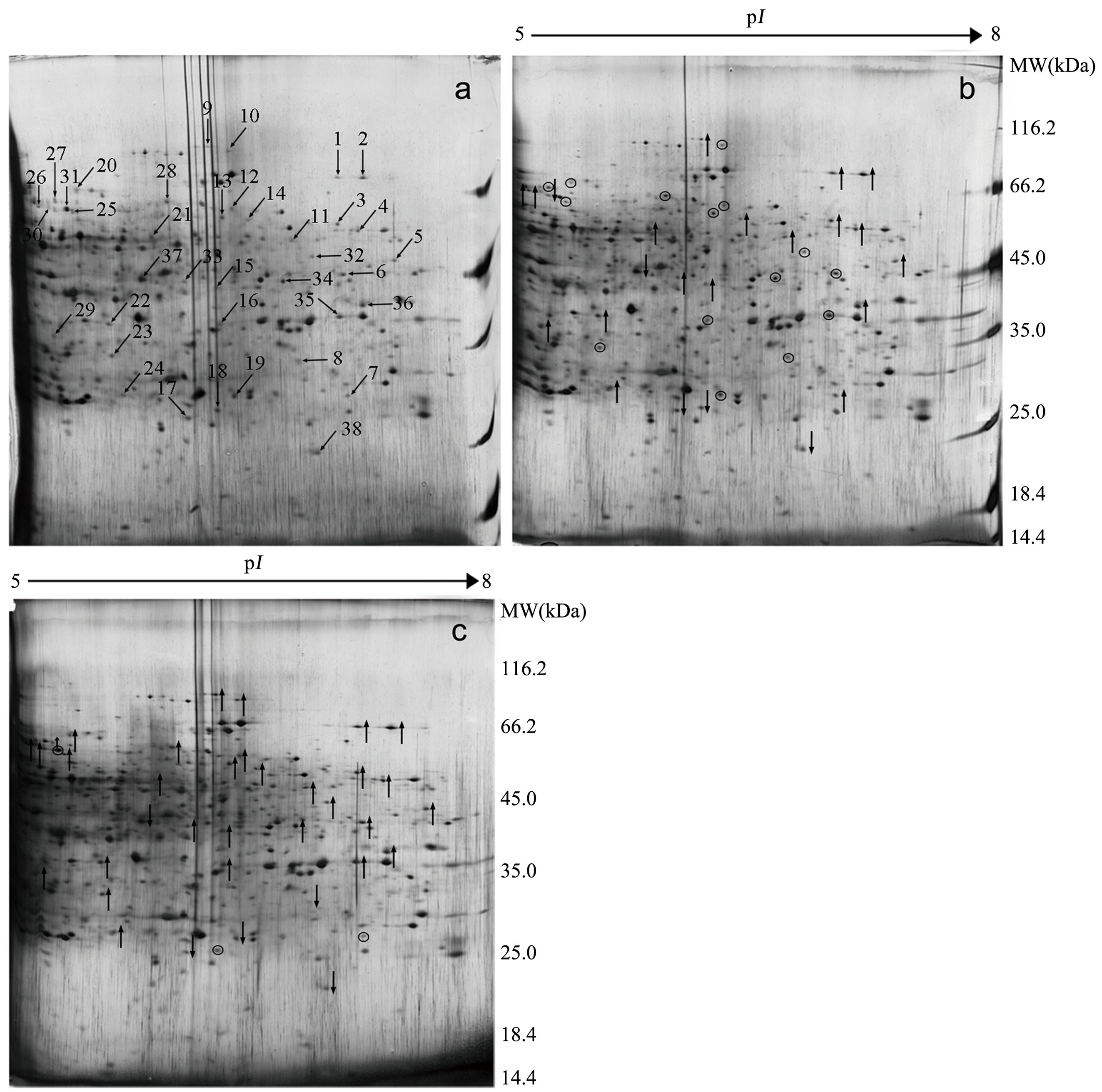


**Figure S1.**

Supplement: Additional file 2: Figure S1 — Silver stained 2-D gel of proteins extracted from the control soil (a), plant cane soil (b) and ratoon cane soil (c). Arrows in (a) point at proteins with differential expression. Upward arrows in (b) and (c) indicate the positions of up-regulated proteins and downward arrows show the positions of down-regulated proteins, while white circles in (b) and (c) represent the same expression level compared to the control. [file 1471-2180-13-135-S2.doc]

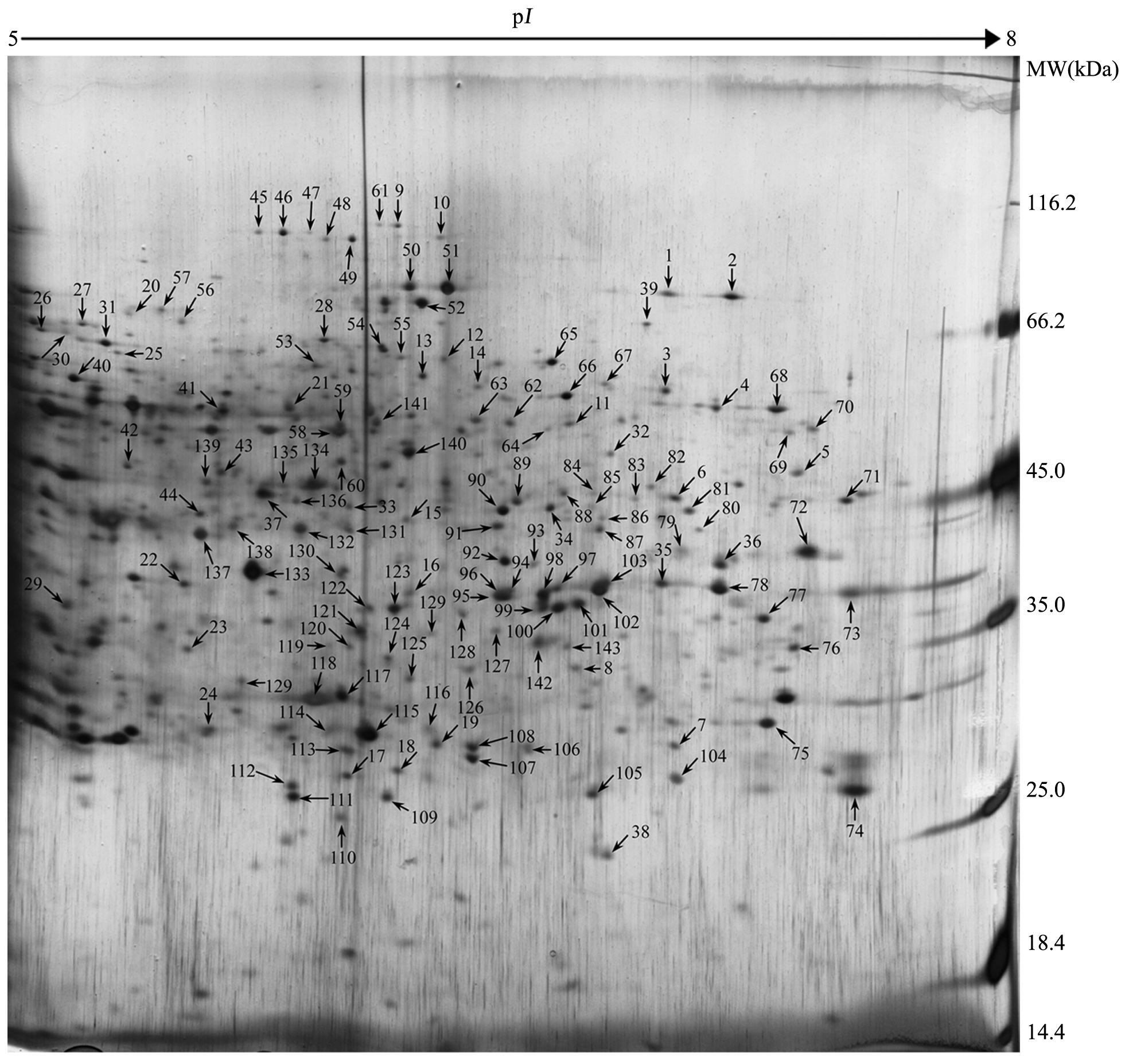


**Figure S2.**

Supplement: Additional file 3: Figure S2 — Representative 2-DE gel of proteins extracted from the plant cane soil. Spot numbers correspond to numbers used in Additional file 4: Table S2. [file 1471-2180-13-135-S3.doc]

**
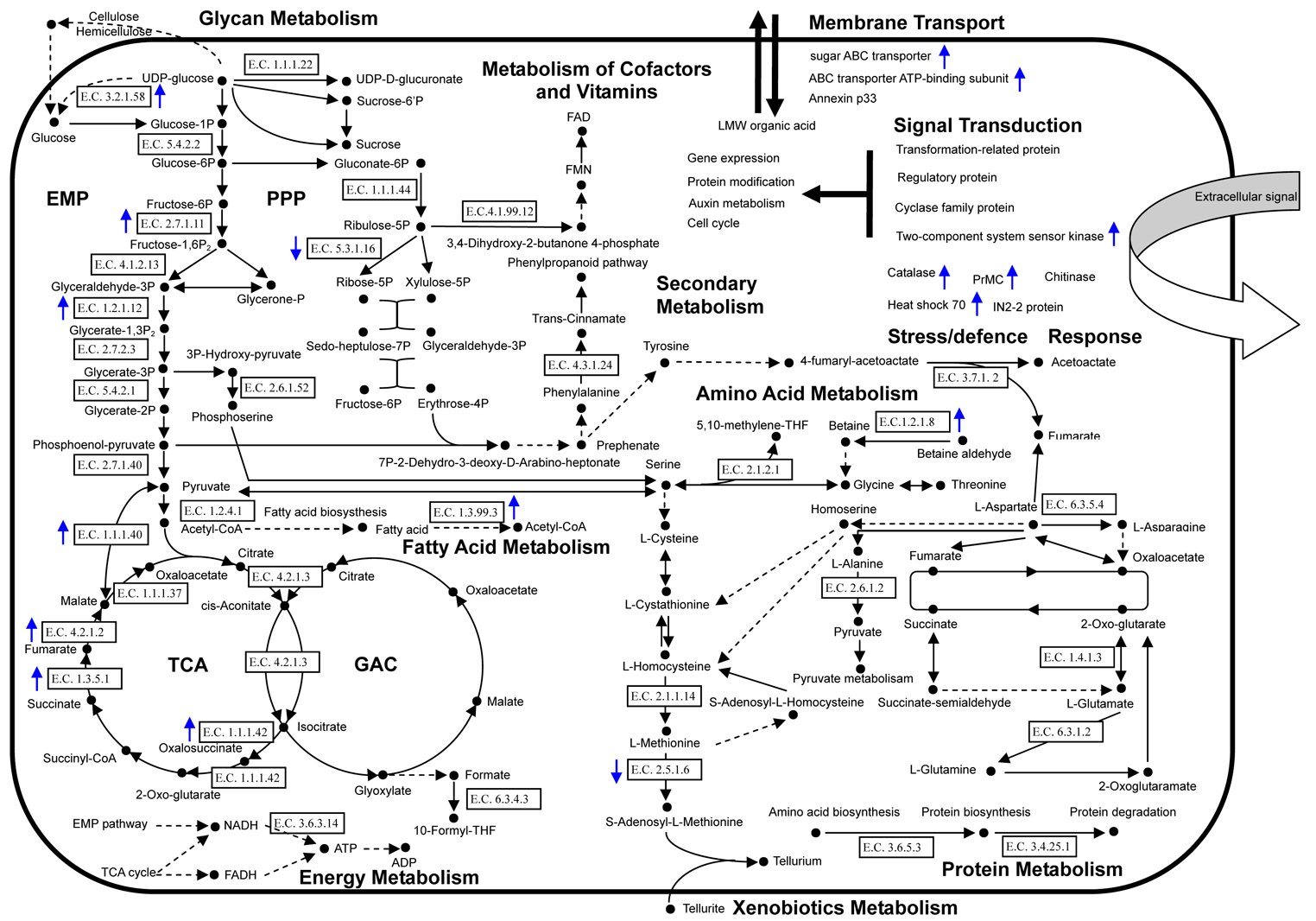
**

**Figure S3.**

Supplement: Additional file 5: Figure S3 — Proposed metabolic model for rhizosphere soil proteins as inferred by metaproteomic data. Identification numbers (E.C.-.-.-.-.) refer to the identified proteins. Blue Upward arrows indicate the up-regulated proteins and downward arrows show the down-regulated proteins. EMP: Embden-Meyerhof pathway; TCA: tricarboxylic acid cycle; GAC: glyoxylic acid cycle; PPP: pentose phosphate pathway. [file 1471-2180-13-135-S5.doc]
