# Supplementary material for: Metaproteomic analysis of ratoon sugarcane rhizospheric soil
Source: BMC Microbiol. 2013 Jun 17;13:135. doi: 10.1186/1471-2180-13-135 (PMC3687580; doi:10.1186/1471-2180-13-135)
Supplement: Additional file 4: Table S2 — Soil proteins identified by MALDI TOF-TOF MS. [file 1471-2180-13-135-S4.doc]

**Table S2. Soil proteins identified by MALDI TOF-TOF MS**

| Spot no. *^a^*) | GI no. *^b^*) | Protein name (Identification number *^c^*)) | Score (PMF) *^d^*) | PMF/  coverage *^e^*) | MW/p*I ^f^*) | Score  (MS-MS) *^g^*) | Pept *^h^*) | Species | Database *^i^*) | Function *^j^*) |
| --- | --- | --- | --- | --- | --- | --- | --- | --- | --- | --- |
| **12** | gi|115470493 | Succinate dehydrogenase [ubiquinone] flavoprotein subunit, mitochondrial (E.C. 1.3.5.1) | 106 | 20/34% | 69494/6.61 | 185 | 3 | *Oryza sativa* | All entries | Tricarboxylic acid cycle (TCA)^1^ |
| **13** | gi|115467370 | Phosphofructokinase (E.C. 2.7.1.11) | 130 | 18/38% | 61907/6.01 | 251 | 4 | *Oryza sativa* | All entries | Embden-Meyerhof pathway (EMP)^2^ |
| **16** | gi|115459078 | Glyceraldehyde-3-phosphate dehydrogenase, cytosolic 3 (E.C. 1.2.1.12) | 117 | 14/51% | 36921/6.34 | 122 | 2 | *Oryza sativa* | All entries | EMP^3^ |
| **18** | gi|115480019 | Proteasome beta type-1 (E.C. 3.4.25.1) | 136 | 11/50% | 24608/6.43 | 92 | 2 | *Oryza sativa* | All entries | Protein degradation^4^ |
| **23** | gi|51090388 | Putative PrMC3 | 107 | 16/59% | 34540/5.61 | 296 | 3 | *Oryza sativa* | All entries | Stress/defense response^5^ |
| **25** | gi|115111257 | Betaine aldehyde dehydrogenase (E.C. 1.2.1.8) | 86 | 10/31% | 55361/5.29 | 276 | 4 | *Oryza sativa* | All entries | Amino acid metabolism^6^ |
| **26** | gi|115464537 | 2,3-bisphosphoglycerate-independent phosphoglycerate mutase (E.C. 5.4.2.1) | 127 | 20/42% | 61003/5.25 | 361 | 5 | *Oryza sativa* | All entries | EMP^7^ |
| **27** | gi|115448989 | Heat shock 70 kDa protein, mitochondrial precursor | 96 | 19/34% | 73081/5.49 | 456 | 4 | *Oryza sativa* | All entries | Stress/defense response^8, 9^ |
| **28** | gi|54606800 | NADP dependent malic enzyme (E.C. 1.1.1.40) | 84 | 24/37% | 65824/5.79 | 193 | 3 | *Oryza sativa* | All entries | Pyruvate metabolism^10^ |
| **29** | gi|115477952 | Cyclase family protein | 80 | 11/39% | 29792/5.32 | 115 | 2 | *Oryza sativa* | All entries | Signal transduction^11, 12^ |
| **31** | gi|115440691 | 2,3-bisphosphoglycerate-independent phosphoglycerate mutase (E.C. 5.4.2.1) | 189 | 30/50% | 60980/5.42 | 500 | 4 | *Oryza sativa* | All entries | EMP^7^ |
| **32** | gi|108708038 | Fumarate hydratase 1, mitochondrial precursor, putative, expressed (E.C. 4.2.1.2) | 124 | 13/27% | 53991/6.93 | 210 | 4 | *Oryza sativa* | All entries | TCA^13^ |
| **35** | gi|968996 | Glyceraldehyde-3-phosphate dehydrogenase (E.C. 1.2.1.12) | 139 | 14/50% | 36641/6.61 | 379 | 3 | *Oryza sativa* | All entries | EMP^3^ |
| **37** | gi|3024122 | S-adenosylmethionine synthase 2 (E.C. 2.5.1.6) | 100 | 18/60% | 43330/5.60 | 405 | 4 | *Oryza sativa* | All entries | Amino acid metabolism^14^ |
| **39** | gi|51536102 | Putative formate-tetrahydrofolate ligase (E.C. 6.3.4.3) | 86 | 15/25% | 68639/6.55 | 122 | 3 | *Oryza sativa* | All entries | One carbon pool^15^ |
| **40** | gi|110289207 | Chaperonin CPN60-1 | 167 | 23/41% | 67551/6.95 | 204 | 4 | *Oryza sativa* | All entries | Protein folding^16^ |
| **41** | gi|115470967 | 1-pyrroline-5-carboxylate dehydrogenase (E.C. 1.5.1.12) | 109 | 22/46% | 57666/5.98 | 379 | 4 | *Oryza sativa* | All entries | Amino acid metabolism^17^ |
| **42** | gi|75243634 | IAA-amino acid hydrolase ILR1-like 3 | 96 | 13/38% | 44076/5.44 | 319 | 4 | *Oryza sativa* | All entries | IAA metabolism^18^ |
| **66** | [gi|115435028](http://www.matrixscience.com/cgi/protein_view.pl?file=../data/20100121/FtmponeeO.dat&hit=gi|115435028&px=1&ave_thresh=53&_sigthreshold=0.05&_server_mudpit_switch=0.001) | Mitochondrial processing peptidase (E.C. 3.4.24.64) | 121 | 16/39% | 54141/6.65 | 233 | 3 | *Oryza sativa* | All entries | Protein metabolism^19^ |
| **71** | gi|115448935 | Beta 1 subunit of 20S proteasome | 109 | 12/47% | 26371/5.47 | 264 | 3 | *Oryza sativa* | All entries | Protein degradation^20^ |
| **72** | [gi|78099751](http://www.matrixscience.com/cgi/protein_view.pl?file=../data/20100121/FtmponTOt.dat&hit=gi|78099751&px=1&ave_thresh=53&_sigthreshold=0.05&_server_mudpit_switch=0.001) | Fructose-bisphosphate aldolase cytoplasmic isozyme (E.C. 4.1.2.13) | 100 | 15/38% | 39238/6.96 | 404 | 4 | *Oryza sativa* | All entries | EMP^21^ |
| **78** | [gi|968996](http://www.matrixscience.com/cgi/protein_view.pl?file=../data/20100106/FtmmSxYeT.dat&hit=gi|968996&px=1&ave_thresh=53&_sigthreshold=0.05&_server_mudpit_switch=0.001) | Glyceraldehyde-3-phosphate dehydrogenase (E.C. 1.2.1.12) | 161 | 16/54% | 36641/6.61 | 594 | 5 | *Oryza sativa* | All entries | EMP^3^ |
| **79** | gi|968996 | Glyceraldehyde-3-phosphate dehydrogenase (E.C. 1.2.1.12) | 139 | 14/50% | 36641/6.61 | 379 | 3 | *Oryza sativa* | All entries | EMP^3^ |
| **87** | gi|115448577 | Pyruvate dehydrogenase E1 alpha subunit (E.C. 1.2.4.1) | 97 | 12/28% | 43017/7.64 | 193 | 4 | *Oryza sativa* | All entries | EMP, TCA^22^ |
| **90** | gi|115455879 | Glutamate dehydrogenase (E.C. 1.4.1.3) | 115 | 14/42% | 44599/6.15 | 168 | 3 | *Oryza sativa* | All entries | Amino acid metabolism^23^ |
| **102** | [gi|121333](http://www.matrixscience.com/cgi/protein_view.pl?file=../data/20100106/FtmmSxEOL.dat&hit=gi|121333&px=1&ave_thresh=52&_sigthreshold=0.05&_server_mudpit_switch=0.001) | Glutamine synthetase root isozyme A (E.C. 6.3.1.2) | 122 | 15/53% | 39435/6.12 | 122 | 3 | *Oryza sativa* | All entries | Amino acid metabolism^24^ |
| **107** | [gi|108706511](http://www.matrixscience.com/cgi/master_results.pl?file=../data/20100106/FtmmSxTSO.dat#Hit1) | Proteasome subunit alpha type 6 (E.C. 3.4.25.1) | 130 | 13/43% | 32472/7.05 | 196 | 3 | *Oryza sativa* | All entries | Protein degradation^25^ |
| **137** | [gi|115459078](http://www.matrixscience.com/cgi/protein_view.pl?file=../data/20100106/FtmmSxene.dat&hit=gi|115459078&px=1&ave_thresh=53&_sigthreshold=0.05&_server_mudpit_switch=0.001) | Glyceraldehyde-3-phosphate dehydrogenase (E.C. 1.2.1.12) | 164 | 20/64% | 36921/6.34 | 369 | 3 | *Oryza sativa* | All entries | EMP^3^ |
| **140** | gi|115465974 | Cytosolic 6-phosphogluconate dehydrogenase (E.C. 1.1.1.44) | 109 | 13/37% | 52973/5.85 | 165 | 2 | *Oryza sativa* | All entries | Pentose phosphate pathway ([PPP](http://themedicalbiochemistrypage.org/pentose-phosphate-pathway.html))^26^ |
| *1* | gi|1203832 | Beta-D-glucan exohydrolase, isoenzyme ExoII (E.C. 3.2.1.58) |  |  | 67835/7.96 | 153 | 2 | *Hordeum vulgare* | All entries | Glycan metabolism^27^ |
| *4* | gi|3868754 | Catalase (E.C. 1.11.1.6) |  |  | 57052/6.49 | 147 | 2 | *Oryza sativa* | All entries | Stress/defense response^28^ |
| *21* | gi|115455455 | UDP-glucose 6-dehydrogenase (E.C. 1.1.1.22) |  |  | 53435/5.79 | 208 | 3 | *Oryza sativa* | All entries | Glycan metabolism^29^ |
| *33* | gi|38605779 | NAD-dependent isocitrate dehydrogenase (E.C. 1.1.1.41) |  |  | 36882/5.77 | 221 | 3 | *Oryza sativa* | All entries | TCA^30^ |
| *43* | gi|115444831 | Fumarylacetoacetase (E.C. 3.7.1.2) |  |  | 47645/5.62 | 112 | 2 | *Oryza sativa* | All entries | Amino acid metabolism^31^ |
| *46* | [gi|75225211](http://www.matrixscience.com/cgi/protein_view.pl?file=../data/20091207/FtmomnYeR.dat&hit=gi|75225211&px=1&ave_thresh=51&_sigthreshold=0.05&_server_mudpit_switch=0.001) | Putative aconitate hydratase (E.C. 4.2.1.3) |  |  | 98591/5.67 | 136 | 2 | *Oryza sativa* | All entries | TCA,Glyoxylic acid cycle (GAC)^32^ |
| *48* | [gi|115450595](http://www.matrixscience.com/cgi/protein_view.pl?file=../data/20100121/FtmporuTt.dat&hit=gi|115450595&px=1&ave_thresh=53&_sigthreshold=0.05&_server_mudpit_switch=0.001) | Aconitate hydratase, cytoplasmic (E.C. 4.2.1.3) |  |  | 106862/6.45 | 307 | 5 | *Oryza sativa* | All entries | TCA, GAC^32^ |
| *50* | [gi|108862990](http://www.matrixscience.com/cgi/protein_view.pl?file=../data/20091207/FtmomnSST.dat&hit=gi|108862990&px=1&ave_thresh=51&_sigthreshold=0.05&_server_mudpit_switch=0.001) | 5-methyltetrahydropteroyltriglutamate-homocysteine methyltransferase, putative, expressed (E.C. 2.1.1.14) |  |  | 79272/7.19 | 352 | 4 | *Oryza sativa* | All entries | Amino acid metabolism^33^ |
| *51* | [gi|108862992](http://www.matrixscience.com/cgi/protein_view.pl?file=../data/20100126/FtmpSfEwT.dat&hit=gi|108862992&px=1&ave_thresh=53&_sigthreshold=0.05&_server_mudpit_switch=0.001) | 5-methyltetrahydropteroyltriglutamate-homocysteine methyltransferase, putative, expressed (E.C. 2.1.1.14) |  |  | 84925/5.93 | 330 | 3 | *Oryza sativa* | All entries | Amino acid metabolism^33^ |
| *53* | gi|115483568 | Pyruvate kinase isozyme G, chloroplast (E.C. 2.7.1.40) |  |  | 61834/6.01 | 317 | 4 | *Oryza sativa* | All entries | EMP^34^ |
| *56* | gi|115454931 | Similar to Phosphoglucomutase, cytoplasmic 2 (E.C. 5.4.2.2) |  |  | 63138/5.40 | 85 | 2 | *Oryza sativa* | All entries | EMP, PPP^35^ |
| *58* | [gi|89280711](http://www.matrixscience.com/cgi/protein_view.pl?file=../data/20091222/FtmmfaTSO.dat&hit=gi|89280711&px=1&ave_thresh=53&_sigthreshold=0.05&_server_mudpit_switch=0.001) | ATP synthase F0 subunit 1 (E.C. 3.6.3.14) |  |  | 55532/5.85 | 417 | 4 | *Oryza sativa* | All entries | Oxidative phosphorylation^36^ |
| *59* | [gi|89280711](http://www.matrixscience.com/cgi/protein_view.pl?file=../data/20091212/FtmolnaSe.dat&hit=gi|89280711&px=1&ave_thresh=53&_sigthreshold=0.05&_server_mudpit_switch=0.001) | ATP synthase F0 subunit 1 (E.C. 3.6.3.14) |  |  | 55532/5.85 | 264 | 3 | *Oryza sativa* | All entries | Oxidative phosphorylation^36^ |
| *63* | [gi|14018051](http://www.matrixscience.com/cgi/protein_view.pl?file=../data/20091212/FtmolnaOR.dat&hit=gi|14018051&px=1&ave_thresh=53&_sigthreshold=0.05&_server_mudpit_switch=0.001) | Putative alanine aminotransferase (E.C. 2.6.1.2) |  |  | 53229/6.23 | 141 | 3 | *Oryza sativa* | All entries | Amino acid metabolism^37^ |
| *81* | [gi|115450835](http://www.matrixscience.com/cgi/protein_view.pl?file=../data/20091223/FtmmrnHSS.dat&hit=gi|115450835&px=1&ave_thresh=53&_sigthreshold=0.05&_server_mudpit_switch=0.001) | Phosphoserine aminotransferase, chloroplast (E.C. 2.6.1.52) |  |  | 45302/8.53 | 98 | 2 | *Oryza sativa* | All entries | Amino acid metabolism^38^ |
| *91* | [gi|14018051](http://www.matrixscience.com/cgi/protein_view.pl?file=../data/20091212/Ftmolnawm.dat&hit=gi|14018051&px=1&ave_thresh=52&_sigthreshold=0.05&_server_mudpit_switch=0.001) | Putative alanine aminotransferase (E.C. 2.6.1.2) |  |  | 53229/6.23 | 210 | 4 | *Oryza sativa* | All entries | Amino acid metabolism^37^ |
| *109* | gi|125527970 | Quinone reductase 2 |  |  | 21749/5.86 | 289 | 3 | *Oryza sativa* | All entries | Stress response^39^ |
| *110* | gi|115474739 | Flavoprotein wrbA |  |  | 21576/6.08 | 226 | 3 | *Oryza sativa* | All entries | Stress response^40^ |
| *117* | [gi|115463789](http://www.matrixscience.com/cgi/protein_view.pl?file=../data/20091206/FtmoobuER.dat&hit=gi|115463789&px=1&ave_thresh=53&_sigthreshold=0.05&_server_mudpit_switch=0.001) | Fructose-bisphosphate aldolase (E.C. 4.1.2.13) |  |  | 36665/6.56 | 139 | 2 | *Oryza sativa* | All entries | EMP^41^ |
| *118* | [gi|54291729](http://www.matrixscience.com/cgi/protein_view.pl?file=../data/20100122/FtmpoxTae.dat&hit=gi|54291729&px=1&ave_thresh=54&_sigthreshold=0.05&_server_mudpit_switch=0.001) | Putative chitinase (E.C. 3.2.1.14) |  |  | 32757/6.08 | 121 | 3 | *Oryza sativa* | All entries | Stress/defense response^42, 43^ |
| *123* | gi|57337458 | Putative malate dehydrogenase (E.C. 1.1.1.37) |  |  | 30653/9.04 | 66 | 2 | *Orpinomyces* | Fungi | TCA^44^ |
| *128* | [gi|115457788](http://www.matrixscience.com/cgi/protein_view.pl?file=../data/20091206/FtmoobuOS.dat&hit=gi|115457788&px=1&ave_thresh=53&_sigthreshold=0.05&_server_mudpit_switch=0.001) | IN2-2 protein |  |  | 38495/6.03 | 128 | 2 | *Oryza sativa* | All entries | Stress/defense response^45^ |
| *132* | [gi|3646373](http://www.matrixscience.com/cgi/protein_view.pl?file=../data/20091203/FtmoracmL.dat&hit=gi|3646373&px=1&ave_thresh=53&_sigthreshold=0.05&_server_mudpit_switch=0.001) | RGP1 protein |  |  | 40079/8.21 | 193 | 3 | *Oryza sativa* | All entries | Glycan metabolism^46^ |
| *133* | [gi|115482534](http://www.matrixscience.com/cgi/protein_view.pl?file=../data/20091223/FtmmrGHwm.dat&hit=gi|115482534&px=1&ave_thresh=52&_sigthreshold=0.05&_server_mudpit_switch=0.001) | Cytosolic malate dehydrogenase (E.C. 1.1.1.40) |  |  | 35888/5.75 | 172 | 2 | *Oryza sativa* | All entries | Pyruvate metabolism^44^ |
| *138* | [gi|2218152](http://www.matrixscience.com/cgi/protein_view.pl?file=../data/20091212/FtmolnatS.dat&hit=gi|2218152&px=1&ave_thresh=52&_sigthreshold=0.05&_server_mudpit_switch=0.001) | Type IIIa membrane protein cp-wap13 |  |  | 40081/6.24 | 89 | 2 | *Vigna unguiculata* | All entries | Glycan metabolism^47^ |
| *139* | [gi|114386664](http://www.matrixscience.com/cgi/protein_view.pl?file=../data/20091223/FtmmrnHet.dat&hit=gi|114386664&px=1&ave_thresh=53&_sigthreshold=0.05&_server_mudpit_switch=0.001) | Phosphoglycerate kinase (E.C. 2.7.2.3) |  |  | 42224/5.64 | 103 | 2 | *Oryza sativa* | All entries | EMP^48^ |
| 2 | gi|226357624 | Putative sugar ABC transporter, periplasmic component (E.C. 3.6.3.17) | 84 | 10/33% | 46676/9.68 |  |  | *Deinococcus deserti* | Bacteria | Membrane transport^49^ |
| 3 | gi|241957693 | Mitochondrial N-glycosylase/DNA lyase (E.C. 4.2.99.18) | 74 | 11/39% | 40573/8.46 |  |  | *Candida dubliniensis* | Fungi | Nucleotide metabolism^50^ |
| 5 | gi|254399905 | ABC transporter ATP-binding subunit | 82 | 18/31% | 66963/5.53 |  |  | *Streptomyces sviceus* | Bacteria | Membrane transport^51^ |
| 6 | gi|126662203 | Oxidoreductase | 74 | 13/20% | 76867/8.83 |  |  | *Flavobacteria bacterium* | Bacteria | Oxidation reduction^52^ |
| 7 | gi|261195979 | ORP1 | 74 | 10/39% | 36747/9.48 |  |  | *Ajellomyces dermatitidis* | Fungi | Signal transduction^53^ |
| 8 | gi|238481813 | ADP-ribosylglycohydrolase (E.C. 3.2.2.24) | 84 | 18/28% | 49119/6.02 |  |  | *Aspergillus flavus* | All entries | Signal transduction^54^ |
| 9 | gi|261854741 | Phosphoribosylformimino-5-aminoimidazole carboxamide ribotide isomerase (E.C. 5.3.1.16) | 85 | 9/41% | 26805/4.63 |  |  | *Halothiobacillus neapolitanus* | Bacteria | Amino acid metabolism^55^ |
| 10 | gi|115456914 | Elongation factor EF-2 (E.C. 3.6.5.3) | 101 | 23/31% | 94939/5.85 |  |  | *Oryza sativa* | All entries | Protein metabolism^56^ |
| 11 | gi|219667596 | Radical SAM domain protein | 82 | 11/46% | 38272/5.24 |  |  | *Desulfitobacterium hafniense* | Bacteria | Diverse reaction^57^ |
| 14 | gi|111024023 | Acyl-CoA dehydrogenase (E.C. 1.3.99.3) | 87 | 13/37% | 41071/5.40 |  |  | *Rhodococcus jostii* | Bacteria | Amino acid metabolism^58^ |
| 15 | gi|23009750 | Succinate dehydrogenase/fumarate reductase, Fe-S protein subunit (E.C. 1.3.5.1) | 87 | 7/92% | 6114/4.52 |  |  | *Magnetospirillum magnetotacticum* | Bacteria | TCA^1^ |
| 17 | gi|253988359 | Phosphoglycerate kinase (E.C. 2.7.2.3) | 83 | 9/33% | 41652/5.19 |  |  | *Photorhabdus asymbiotica* | Bacteria | EMP^59^ |
| 19 | gi|94497581 | Electron-transferring-flavoprotein dehydrogenase (E.C. 1.5.5.1) | 84 | 9/25% | 61194/5.66 |  |  | *Sphingomonas* sp. | All entries | Energy metabolism^60^ |
| 20 | gi|85110870 | Related to kinesin-like protein | 83 | 9/33% | 41652/5.19 |  |  | *Photorhabdus asymbiotica* | Bacteria | Cytoskeleton protein^61^ |
| 22 | gi|194366013 | Nitrate reductase, alpha subunit (E.C. 1.7.1.1) | 71 | 19/16% | 140507/5.98 |  |  | *Stenotrophomonas maltophilia* | All entries | Nitrogen metabolism^62^ |
| 24 | gi|21492793 | Conjugal transfer protein A | 91 | 24/19% | 171793/6.93 |  |  | *Rhizobium etli* | All entries | Bacterial conjugation^63^ |
| 30 | gi|219664364 | Two-component system sensor kinase (E.C. 2.7.13.3) | 87 | 19/15% | 176010/6.50 |  |  | *Rhodococcus* sp. | Bacteria | Signal transduction^64^ |
| 34 | gi|126135008 | Isocitrate dehydrogenase [NADP], mitochondrial precursor (E.C. 1.1.1.42) | 76 | 14/32% | 48355/8.21 |  |  | *Pichia stipitis* | Fungi | TCA^65^ |
| 36 | gi|52426030 | MrcA protein | 90 | 18/25% | 96552/6.40 |  |  | *Mannheimia succiniciproducens* | Bacteria | Glycan metabolism^66^ |
| 38 | gi|148685933 | Tubulin, gamma complex associated protein 2, isoform CRA_a | 90 | 18/29% | 89598/6.52 |  |  | *Mus musculus* | All entries | Cytoskeleton protein^67^ |
| 44 | gi|227981206 | Sulfite reductase, beta subunit (hemoprotein) (E.C. 1.8.1.2) | 82 | 14/20% | 64464/5.49 |  |  | *Tsukamurella paurometabola* | Bacteria | Sulfur metabolism^68^ |
| 45 | gi|126132366 | Cell division control protein 48 | 74 | 11/29% | 91508/4.84 |  |  | *Pichia stipitis* | Fungi | Signal transduction^69^ |
| 47 | gi|262038161 | OstA family protein | 84 | 19/27% | 81455/9.21 |  |  | *Leptotrichia goodfellowii* | Bacteria | Stress response^70^ |
| 49 | gi|189200336 | Sporulation protein | 72 | 17/23% | 112116/6.29 |  |  | *Pyrenophora tritici-repentis* | Fungi | Signal transduction^71^ |
| 52 | gi|115447403 | Phenylalanine ammonia-lyase (E.C. 4.3.1.24) | 135 | 14/26% | 76021/6.07 |  |  | *Oryza sativa* | All entries | Secondary metabolism^72^ |
| 61 | gi|126662003 | Asparagine synthetase B (E.C. 6.3.5.4) | 80 | 15/27% | 61277/5.61 |  |  | *Flavobacteria bacterium* | Bacteria | Amino acid metabolism^73^, Nitrogen metabolism^74^ |
| 67 | gi|107023097 | ATP/GTP-binding protein | 84 | 11/52% | 34655/5.80 |  |  | *Burkholderia cenocepacia* | Bacteria | Energy metabolism^75^ |
| 68 | [gi|115485405](http://www.matrixscience.com/cgi/protein_view.pl?file=../data/20100121/FtmponsTO.dat&hit=2) | Serine hydroxymethyltransferase (E.C. 2.1.2.1) | 78 | 18/36% | 51799/7.16 |  |  | Oryza sativa | All entries | Amino acid metabolism^76^ |
| 73 | gi|255727695 | Mitochondrial ribosomal protein L8 | 78 | 13/63% | 29860/9.25 |  |  | *Candida tropicalis* | Fungi | Mitochondrial protein metabolism^77^ |
| 75 | gi|145234049 | Peptidase S15 | 72 | 7/45% | 17840/10.25 |  |  | *Aspergillus niger* | Fungi | Protein metabolism^78^ |
| 84 | gi|260945048 | G-protein signaling regulator | 73 | 7/29% | 49207/7.25 |  |  | *Clavispora lusitaniae* | Fungi | Signal transduction^79^ |
| 85 | gi|192290036 | ABC transporter ATP-binding protein | 88 | 8/49% | 34941/5.76 |  |  | *Rhodopseudomonas palustris* | Bacteria | Membrane transport^80^ |
| 88 | gi|92112843 | Phosphate transport regulator | 88 | 8/61% | 25660/4.94 |  |  | *Chromohalobacter salexigens* | Bacteria | Signal transduction^81^ |
| 92 | gi|255727695 | Mitochondrial ribosomal protein L8 | 77 | 10/52% | 29860/9.25 |  |  | *Candida tropicalis* | Fungi | Mitochondrial protein metabolism^77^ |
| 93 | gi|255727695 | Mitochondrial ribosomal protein L8 | 82 | 9/45% | 29860/9.25 |  |  | *Candida tropicalis* | Fungi | Mitochondrial protein metabolism^77^ |
| 95 | gi|125548785 | Glyceraldehyde-3-phosphate dehydrogenase (E.C. 1.2.1.12) | 87 | 13/36% | 42368/6.41 |  |  | *Oryza sativa* | All entries | EMP^3^ |
| 96 | gi|170103931 | Glycosyltransferase family 39 protein | 74 | 11/33% | 43350/8.36 |  |  | *Laccaria bicolor* | Fungi | Protein metabolism^82^ |
| 97 | gi|85090893 | 3,4-dihydroxy-2-butanone 4-phosphate synthase (E.C. 4.1.99.12) | 83 | 11/50% | 26075/5.07 |  |  | *Neurospora crassa* | Fungi | Cofactors Metabolism^83^ |
| 99 | gi|108760560 | Patatin domain-containing protein | 94 | 11/74% | 23892/6.66 |  |  | *Myxococcus xanthus* | Bacteria | Virulence factor^84^ |
| 100 | gi|291184583 | Actin patches distal protein 1 | 74 | 11/36% | 33458/6.79 |  |  | *Trichophyton verrucosum* | Fungi | Stress/defense response^85^ |
| 101 | gi|115452789 | Ricin B-related lectin domain containing protein | 150 | 18/54% | 39254/6.30 |  |  | *Oryza sativa* | All entries | Plant resistance^86^ |
| 103 | gi|220914313 | Glutamate dehydrogenase (NADP(+)) | 82 | 15/27% | 48015/5.28 |  |  | *Arthrobacter chlorophenolicus* | Bacteria | Amino acid metabolism^87^ |
| 104 | gi|75762505 | Tellurite resistance protein | 85 | 10/44% | 27371/6.54 |  |  | *Bacillus thuringiensis serovar israelensis* | Bacteria | Xenobiotics metabolism^88^ |
| 112 | gi|255727695 | Mitochondrial ribosomal protein L8 | 89 | 14/63% | 29860/9.25 |  |  | *Candida tropicalis* | Fungi | Mitochondrial protein metabolism^77^ |
| 114 | gi|121601839 | lipoyl synthase (E.C. 2.8.1.8) | 87 | 13/36% | 35937/8.80 |  |  | *Bartonella bacilliformis* | Bacteria | Cofactors Metabolism^89^ |
| 119 | gi|33415065 | Transformation-related protein 16 | 88 | 10/40% | 36477/9.55 |  |  | *Homo sapiens* | All entries | Signal transduction^90^ |
| 120 | gi|149277615 | Putative regulatory protein | 90 | 9/40% | 28085/5.10 |  |  | *Pedobacter* | Bacteria | Transcriptional regulatiion^91^ |
| 124 | gi|255727695 | Mitochondrial ribosomal protein L8 | 86 | 10/52% | 29860/9.25 |  |  | *Candida tropicalis* | Fungi | Mitochondrial protein metabolism^77^ |
| 126 | gi|115467154 | Annexin p33 | 322 | 6/18% | 35984/6.21 |  |  | *Oryza sativa* | All entries | Membrane transport^92^,  Signal transduction^93^ |
| 134 | gi|100801668 | S-adenosylmethionine synthetase (E.C. 2.5.1.6) | 163 | 17/57% | 42979/5.74 |  |  | *Oryza rufipogon* | All entries | Amino acid metabolism^94^ |
| 135 | gi|255711184 | Putative S-adenosylmethionine-dependent methyltransferase of the seven beta-strand family | 78 | 10/36% | 33018/6.61 |  |  | *Lachancea thermotolerans* | Fungi | Amino acid metabolism^95^ |
| 141 | gi|257466736 | Filamentous hemagglutinin outer membrane protein | 85 | 14/47% | 40650/9.16 |  |  | *Fusobacterium gonidiaformans* | Bacteria | Virulence factor^96^ |
| 142 | gi|255727695 | Mitochondrial ribosomal protein L8 | 83 | 12/51% | 29860/9.25 |  |  | *Candida tropicalis* | Fungi | Mitochondrial protein metabolism^77^ |
| 143 | gi|242804562 | Pentalenene synthase (E.C. 4.2.3.7) | 76 | 7/30% | 21332/4.90 |  |  | *Talaromyces stipitatus* | Fungi | Secondary metabolism^97^ |

**Note: ^a)^ The numbering corresponds to the 2-DE gel in Fig. S2. Protein spots in bold shared equal searching by MS/MS and MS. Protein spots with italics matched at least two MS/MS peptides. The remainders matched at least three PMFs. ^b)^ GI number in NCBI. ^c)^ a unique 4-digit identification number for enzyme identification by the Enzyme Commission (E.C.). ^d)^ MASCOT score of PMF. ^e)^ The number of peptides identified by MS/sequence percentage coverage. ^f)^ Theoretical molecular weight and p*I*. ^g)^ MASCOT score of MS/MS. ^h)^ Number of peptides identified by MS/MS. ^i)^ The used database in the process of MASCOT search. ^j)^ Functional classification using KEGG database.**
